# IRF1 is a context-dependent homeostatic gatekeeper of basal immunity and antiviral readiness

**DOI:** 10.1016/j.jbc.2025.111118

**Published:** 2025-12-30

**Authors:** Eyal Zoler, Irina Miodownik, Shifra Ben-Dor, Daniel Harari, Jiri Zahradnik, Ariel Afek, Gideon Schreiber

**Affiliations:** 1 Department of Biomolecular Sciences, Weizmann Institute of Science, Rehovot, Israel; 2 Department of Chemical and Structural Biology, Weizmann Institute of Science, Rehovot, Israel; 3 Bioinformatics Unit, Life Sciences Core Facilities, Weizmann Institute of Science, Rehovot, Israel; 4 First Medical Faculty at Biocev, Charles University, Vestec, Prague, Czechia

**Keywords:** antiviral activity, gene regulation, interferon, IRF1, Transcription factor

## Abstract

Interferon regulatory factor 1 (IRF1) plays a pivotal role in interferon (IFN) signaling. Here, we dissect the impact of IRF1 on gene transcription regulation in HeLa cells, by targeted knockout (KO) or overexpression of IRF1. *IRF1* KO partially diminished IFN-γ but not IFN-β induced gene regulation. *IRF1* KO did show a homeostatic role in basal transcript abundance, including increasing the abundance of antiviral gene transcripts, apparently through increased expression of other IRF genes. IRF1 overexpression induced potent antiviral protection, which is mediated by secretion of type I IFN proteins, particularly of IFN-α subtypes, which expression is driven by IRF1. This paracrine effect was confirmed by transcriptomics, cytokine profiling, and mass spectrometry. Surprisingly, antiviral protection was observed also in *JAK1* KO or ruxolitinib-treated cells but not in type I IFN receptor KO cells, suggesting the involvement of noncanonical signaling pathways. Hierarchical clustering of RNA-seq data revealed distinct IFN-independent gene clusters activated or repressed by IRF1, including pathways related to adaptive immunity and T cell function. Using protein-binding microarrays and predictive modeling, we generated an energy-normalized binding matrix for IRF1, enabling sequence-specific prediction of promoter-binding affinities beyond classical consensus motifs. This approach allows estimation of IRF1-binding potential across diverse genomic contexts as validated for the IFIT2 gene promoter by a reporter assay. Evaluating the biological significance of our study, we show that IRF1 abundance varies by 10,000-fold between cell lines, with positive correlations of IRF1 with the abundance of gene transcripts involved in antiviral and immune-driving activities.

Type I interferons (IFN-I) are essential in initiating both innate and adaptive immune responses against various pathogens (1). They also play a significant role in regulating tumor immunity and contribute to the development of autoimmune diseases. IFN-Is are secreted proteins that induce antiviral activity in almost all nucleated cells in vertebrates, although their antiproliferative and immunomodulatory activities vary with cell type (2, 3, 4). The human IFN-I family comprises 17 members, including 13 subtypes of IFN-α, which exhibit high sequence and structural similarity (5, 6, 7), along with IFN-β, IFN-κ, IFN-ω, and IFN-ε (8, 9). These IFN-I members all utilize a common receptor composed of the subunits IFN-α receptor 1 (IFNAR1) and IFN-α receptor 2 (IFNAR2) (3, 10).

Upon the engagement of IFN-I with its receptor subunits, a ternary complex is formed, triggering the JAK-STAT signaling pathway. This activation results in the phosphorylation of JAKs and key tyrosine residues on the STAT proteins (11, 12), facilitating their dissociation from the receptor, dimerization, and nuclear translocation where, together with the interferon regulatory factor (IRF)9, they function as transcription factors (13, 14). This process significantly alters the expression of thousands of interferon-stimulated genes (ISGs) (15, 16, 17), including a marked upregulation of certain IRFs, especially IRF1 (18).

Interferon-gamma (IFN-γ), which signals through IFNGR, is a crucial cytokine primarily produced by natural killer (NK) cells, activated T cells, and some antigen-presenting cell subsets and is an important component of innate and adaptive immune responses (19, 20, 21). It plays a vital role in immune surveillance by enhancing the antimicrobial functions of macrophages and stimulating antigen presentation. The signaling cascade initiated by IFN-γ binding to its receptor activates the JAK-STAT pathway, culminating in the expression of ISGs, many of which mediate antiviral and antibacterial immunity (22, 23). Among the key transcription factors downstream of IFN-γ signaling is IRF1 (24). IRF1 is induced directly by IFN-γ and serves as a master regulator in the immune response. It enhances the expression of various immune-related genes, including those involved in inflammation, apoptosis, and antigen processing (25, 26, 27).

Discovered in 1988 by Taniguchi, IRF1 was the first identified member of the IRF family, noted for its transcriptional activation in nuclear extracts postviral infection (28). IRF1 is a versatile transcriptional regulator critical for various cellular responses, including the host response to viral and bacterial infections, and its involvement in the expression of numerous genes in hematopoiesis, inflammation, immune responses, and cell cycle control (29, 30, 31). It acts as both a transcriptional activator and repressor (32, 33, 34, 35, 36), binding to interferon-stimulated response elements (ISREs) in the promoters of its target genes (29, 34, 36, 37, 38), thus playing an essential role in IFN signaling, immune regulation, tumor suppression, and responses to genotoxic stress (30, 32).

IRFs play critical roles in orchestrating immune responses to viral infections, with IRF9 and IRF1 being central regulators in the type I and type II interferon pathways, respectively. IRF9 is primarily involved in the type I IFN response. It forms a complex with STAT1 and STAT2, known as ISGF3 (interferon-stimulated gene factor 3), which binds to ISREs on target genes, initiating the transcription of a wide array of ISGs (39, 40, 41, 42). Although IRF9 and IRF1 are associated with distinct interferon pathways, growing evidence highlights their functional overlap and synergy in modulating antiviral immunity and inflammation (43). IRF1 and IRF9 share the ability to bind ISREs, which enables them to regulate overlapping sets of genes (44). While IRF1 can act independently of IRF9, particularly in IFN-γ-driven immune responses, recent studies show that these two transcription factors often act together to amplify the transcriptional activation of key ISGs (43).

There is compelling evidence that IRF1 suppresses the replication of a variety of RNA viruses and plays a critical role in host antiviral defense, although this can vary depending on the cell type and specific virus. For instance, IRF1 is crucial for activating the transcription of type III IFNs in human hepatocytes infected with Sendai virus (45). However, type III IFNs, due to their localized receptor abundance and insufficient STAT1 activation, fail to induce IRF1 expression or activate its proinflammatory gene expression program in epithelial cells. In contrast, the antiviral effects of type I and II IFNs are more robust (46). In addition to its established antiviral role, IRF1 has been implicated in T cell development, linking it to adaptive immunity and cancer immunosurveillance (47, 48, 49). These emerging roles expand the relevance of IRF1 beyond acute infection to tumor immunity and chronic inflammation. Despite this breadth of function, key aspects of IRF1 biology remain unclear, particularly its role in maintaining basal ISG expression and its capacity to function independently of the canonical JAK-STAT pathway. While some studies suggest IRF1 can induce transcription in a STAT-independent manner, the extent and mechanism of this activity are still not sufficiently understood (50).

Similar to other members of the IRF family, IRF1 features an N-terminal DNA-binding domain (DBD) characterized by a sequence of five meticulously conserved tryptophan-rich repeats (51, 52). The IRF1 DBD shows a high affinity for the consensus DNA sequence 5′-GAAANNGAA-3′, where "N" represents any nucleotide. Additionally, it can bind to various nonconsensus sequences, albeit with lower affinity (53). The IRF1 DBD comprises 136 amino acids with a molecular mass of 16 kDa. This compact domain adopts a helix-turn-helix motif, a structural feature commonly found in DNA-binding proteins (54). The helix-turn-helix motif in the IRF1 DBD facilitates its interaction with the DNA backbone, enhancing its specificity for binding ISRE. The IRF1 DBD binds to the promoters of a broad spectrum of genes, including interferons and genes involved in antiviral protection and cell growth regulation (29, 32, 34, 35, 36). The ability of the IRF1 DBD to interact with diverse promoters emphasizes its extensive influence on gene expression and its significance in regulating cellular responses to IFN signaling. In summary, the IRF1 DBD capacity to recognize and bind specific DNA sequences enables IRF1 to orchestrate the transcriptional regulation of IFN-inducible genes, highlighting its crucial role in maintaining cellular health.

Although IRF1 has long been recognized as a transcriptional effector of interferon signaling, recent studies have shown that it also contributes to enhancer activation and chromatin remodeling, cooperating with ISGF3 and GAF to regulate ISG networks (55, 56). Here, we complement this emerging view by quantifying IRF1–DNA-binding affinities, functionally validating predicted promoter sites, and showing that the antiviral response in epithelial cells is a paracrine type I IFN-mediated effect, which partly circumvent the JAK/STAT pathway. Using IRF1 knockout (KO) and overexpression (OE) HeLa cells, we investigate how IRF1 modulates interferon signaling, immune gene regulation, and antiviral responses. As part of this effort, we applied protein-binding microarrays (PBMs) to generate an energy-normalized binding matrix for IRF1, enabling sequence-specific prediction of promoter-binding affinities beyond classical consensus motifs. This approach allows estimation of IRF1-binding potential across diverse genomic contexts, assuming chromatin accessibility. Notably, our study compares both type I and type II IFNs, which are known to drive robust antiviral activity. While IRF1’s involvement in IFN responses is well established, its specific roles in maintaining basal ISG expression, regulating cytokine output, and operating independently of canonical JAK-STAT signaling remain incompletely defined. Elucidating these context-specific functions is particularly relevant given IRF1’s emerging importance in immune homeostasis and its potential therapeutic relevance in cancer and infection settings where IFN signaling may be impaired.

## Results

### Loss of IRF1 disrupts interferon signaling homeostasis and promotes constitutive antiviral pathway activation

**Figure 1:**
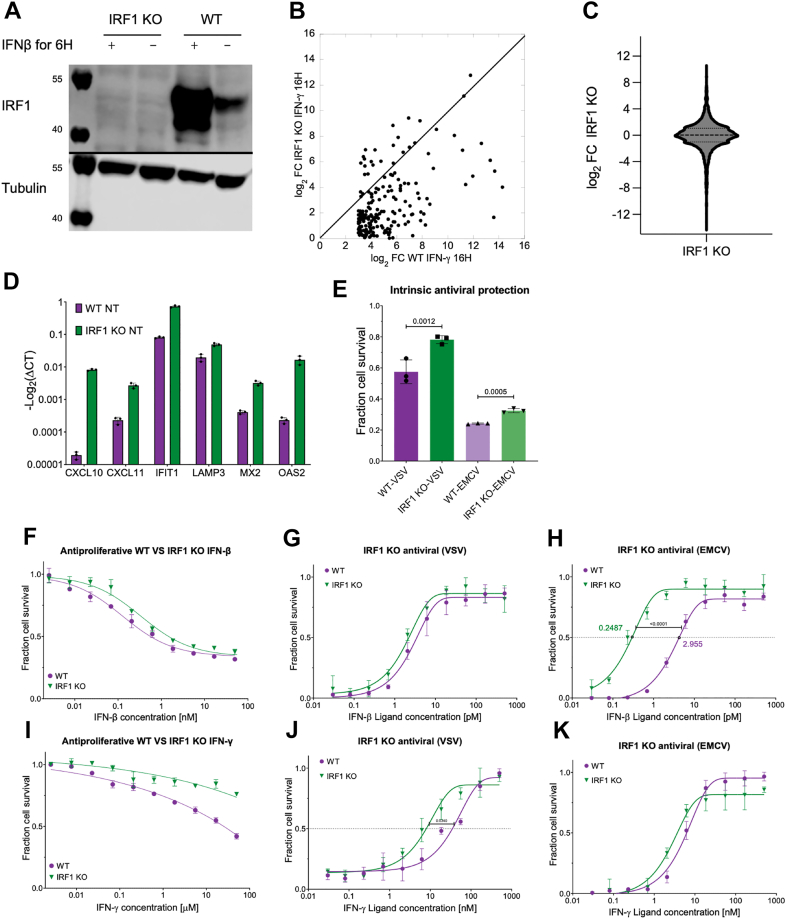
**Characterization of *IRF1* KO HeLa cells.***A*, Western blot analysis of IRF1 protein abundance in WT and IRF1 KO HeLa cells. Quantification of three replicates of this blot is presented in Figure S1*D*. *B*, gene transcript abundance (relative to nontreated) of *IRF1* KO HeLa cells plotted against WT cells following 16 h treatment with IFN-γ (100 nM). *C*, gene transcript abundance of nontreated *IRF1* KO HeLa cells relative to WT. *D*, gene transcript abundance of selected ISGs in WT and *IRF1* KO cells, measured by qRT-PCR. Data are shown as –log_2_(ΔCT) values normalized to HPRT1. Genes include robust (XAF1, MX1, MX2, and OAS2) and tunable ISGs (IDO1, CXCL10, and CXCL11). Data represent mean ± SD of three independent experiments. *E*, intrinsic antiviral protection of WT and *IRF1* KO cells following infection with VSV or EMCV in the absence of exogenous IFN. Statistical significance was assessed by one-way ANOVA followed by Tukey’s *post hoc* test*. F* and *I*, antiproliferative potency of IFN-β (*F*) or IFN-γ (*I*). Cells were treated for 96 h and stained with crystal violet to assess viability. *G* and *H*, antiviral activity of WT and *IRF1* KO cells treated with IFN-β for 4 h prior to VSV (18 h) (*G*) or EMCV (20 h) infection (*H*). *J* and *K*, is like (*G* and *H*) but treated with IFN-γ for 8 h prior to VSV (18 h) (*J*) or EMCV (20 h) (*K*) infection. Cell viability was determined by crystal violet staining. Data represent median of 3 to 5 independent experiments; error bars indicate SD and statistical significance was assessed using a two-tailed *t* test. EMCV, encephalomyocarditis virus; HPRT1, hypoxanthine-guanine phosphoribosyltransferase 1; IFN, interferon; IRF1, interferon regulatory factor 1; ISG, interferon-stimulated gene; KO, knockout; VSV, vesicular stomatitis virus.

We have previously shown that *STAT2* KO results in the loss of induction of gene transcription driven by ISRE promotor elements, while genes driven by GAS promotor elements remained unaffected. Only a dual KO of *STAT2* and *IRF1* fully inhibited type I IFN signaling (11). Indeed, IRF1 has been found to be essential for activation of a number of GAS-mediated genes (57). To further delineate the specific role of IRF1, we generated an *IRF1* KO HeLa cell line. We assumed that the loss of IRF1 would impair the upregulation of gene expression driven by the GAS promoter activated by STAT1 homodimers, while STAT2 KO impairs type I IFN-induced ISGF3 formation. Western blot (WB) analysis confirmed the absence of IRF1 protein independent of IFN-β treatment (Fig. 1*A*). *IRF1* KO did not significantly impact transcript abundance following IFN-β treatment (Fig. S1*A*); however, levels of transcript abundance following IFN-γ treatment were lower for many (but not all) IFN-γ induced genes (Fig. 1*B*). Interestingly, *IRF1* KO altered transcript abundance in nontreated cells, as determined by real-time PCR (Fig. 1*D*) and RNA-seq (Fig. 1*C*). RNA-seq suggested higher basal abundance of several IRFs, including *IRF7* and *IRF9* (Fig. S1, *B* and *C*). To validate that increased mRNA abundance translate to protein abundance of IRF7 and IRF9 proteins, we performed WB, showing a similar trend (Fig. S1, *E* and *F*).

Given the substantial alterations observed in basal transcript abundance, we performed an antiviral assay using a short viral incubation time without IFN treatment (see Experimental procedures, Intrinsic Antiviral Protection). Under these conditions, *IRF1* KO cells exhibited enhanced resistance to both vesicular stomatitis virus (VSV) and encephalomyocarditis virus (EMCV) (Fig. 1*E*), likely attributable to the elevated basal abundance of key ISGs. Next, we monitored the effect of *IRF1* KO on IFN-β- and IFN-γ-induced antiproliferative and antiviral potency, showing no effect for the former and only a modest change in the antiproliferative activity upon IFN-γ treatment (Fig. 1, *F* and *I*). Following, we assessed the antiviral activity of *IRF1* KO cells against VSV and EMCV, after IFN-β and IFN-γ treatments (Fig. 1, *G*, *H*, *J* and *K*). The differences between the WT and KO cells were subtle; however, we observed a slight decrease in the EC50 values in the KO cells, which was statistically significant only for EMCV treated with IFN-β (Fig. 1*H*). Our hypothesis for the subtle differences between the *IRF1* KO and wildtype cells is that the KO exhibits compensatory increased abundance of other IRFs, as shown in Fig. S1, *E* and *F*.

**Figure 2:**
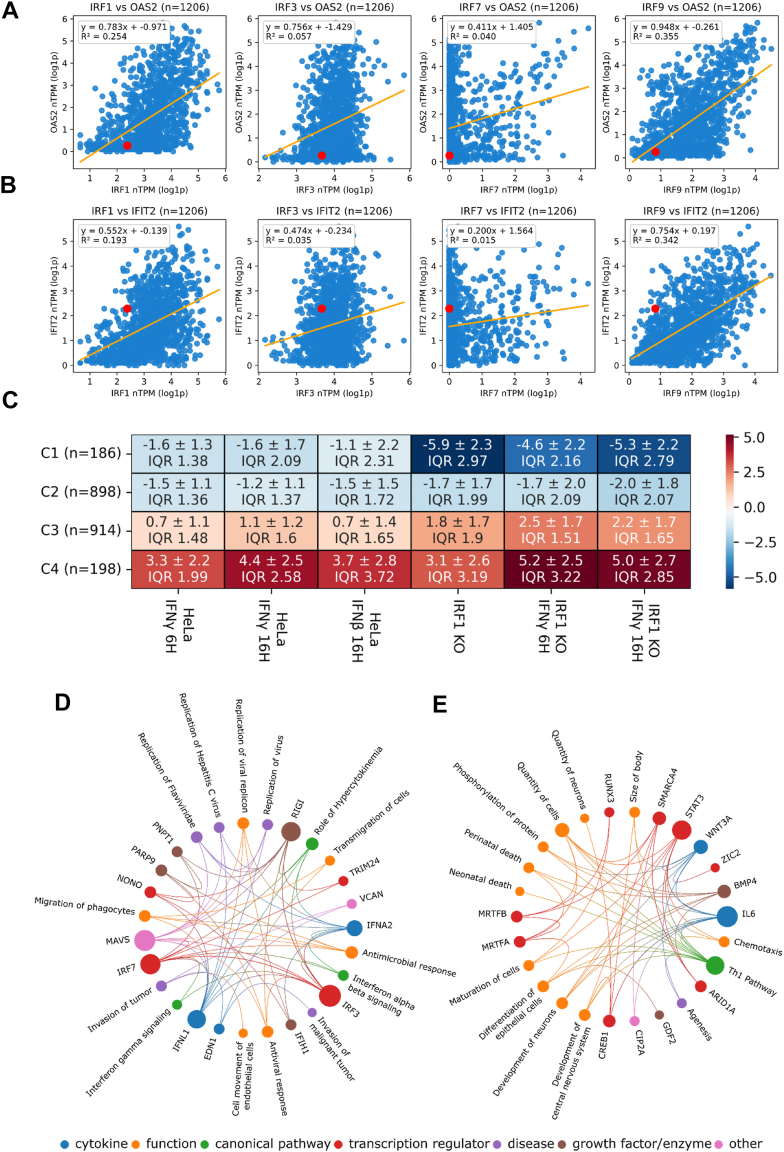
**Transcriptomic analysis of *IRF1* KO cells, gene expression patterns and pathway analysis.***A* and *B*, transcription abundance correlation between *OAS2* (*A*) and *IFIT1* (*B*) and *IRF1, IRF3, IRF7, and IRF9* across 1206 human cell types. Data are from the Human Protein Atlas (https://www.proteinatlas.org/about/download). Scatter plots show log_1__0_-transformed normalized Transcripts Per Million (nTPM) values (log1p). *Blue dots* represent individual cell types; HeLa is highlighted in *red*. Linear regression was performed for each gene pair, and R^2^ values are shown in each panel. *C*, heatmap showing hierarchical clustering of genes with significant expression changes (|log2(FC)| > 2) relative to nontreated WT HeLa cells across the following conditions: WT cells treated with IFN-γ (100 nM) for 6 h, WT cells treated with IFN-γ (100 nM) for 16 h, WT cells treated with IFN-β (2 nM) for 16 h, *IRF1* KO cells, *IRF1* KO cells treated with IFN-γ (100 nM) for 6 h, and *IRF1* KO cells treated with IFN-γ (100 nM) for 16 h. Clusters are divided into four groups based on median expression, with standard deviation and interquartile range (IQR) calculated for each group. *D* and *E*, pathway analysis of Cluster 4 (*D*) and Cluster 1 (*E*), performed using QIAGEN Ingenuity Pathway Analysis (IPA). Pathways were selected based on a z-score > 2 and a *p*-value of overlap < 0.05, indicating significant pathway enrichment in genes which expression was upregulated in response to *IRF1* KO and IFN treatments. IFN, interferon; IRF1, interferon regulatory factor 1; KO, knockout.

To evaluate whether abundance of IRF1 relates to abundance of other gene transcripts in multiple different cell types, we analyzed large-scale RNA-seq data from 1206 human cell types using the Human Protein Atlas (58). Violin plot analysis of ISG abundance ratios across these cell types revealed that abundance of IRF1 in relation to other ISGs is near the global median for HeLa cells (Fig. S2*A*). Next, we performed correlation analyses of IRF1 in relation to other IRFs and representative ISGs. IRF1 abundance correlates with IRF9 but not IRF3 or IRF7. In addition, good positive associate is found with key ISGs such as interferon-induced protein with tetratricopeptide repeats 2 (IFIT2), IFIT1, and OAS2 across the cell atlas (Figs. 2, *A* and *B*, and S2*B*). In particular, high IRF1 and IRF9 expression were consistently linked to higher ISG expression, reinforcing their role in maintaining basal transcript abundance of these ISGs. While these findings help contextualize our HeLa-based observations, it should be noted that cell type specificity in both the homeostatic and induced IFN responses have been often observed (59).

To gain detailed insight into the transcriptomic changes, we further analyzed our RNA-seq data, comparing WT and *IRF1* KO cells, untreated (NT) or treated with IFN-γ for 6 h and 16 h or IFN-β for 16 h. WT NT cells served as the baseline for fold-change (FC) analysis. Using the UTAP (Universal Transcriptome Analysis Pipeline) (60) and the DESeq2 package (61), we identified significant transcriptomic alterations in the absence of IRF1 (Figs. 2, *C*–*E* and S1*B*). A volcano plot of NT *IRF1* KO cells (Fig. S1*B*) revealed higher abundance of key antiviral genes such as *MX1*, *IFIT3*, and *IL6*, alongside lower abundance of genes like *AQP3* and *LPL* (see also Fig. S3). These findings suggest that IRF1 plays a crucial role in maintaining the basal abundance of some IFN-regulated genes. Next, we organized the data into a heat map, arranged by hierarchical clustering, which was divided into four distinct groups (Fig. 2*C*). This approach helped us visualize the gene transcript abundance changes and categorize them. Among the clusters, two stood out as particularly interesting. Cluster 1 constitutes genes whose abundance is marginally reduced by treatment with IFNβ or IFNγ but is strongly decreased in *IRF1* KO cells (independent of IFN treatment), with a median log_2_-fold change of approximately −5. Cluster 4 constitutes genes whose abundance is increased in *IRF1* KO cells both without and after IFN treatment, with a median log_2_-fold change of 3 to 5. To further elucidate the functional significance of these gene clusters, we performed pathway analysis using QIAGEN Ingenuity Pathway Analysis (IPA) (62) for cluster 4 (Fig. 2*D*) and cluster 1 (Fig. 2*E*). As anticipated from the IFN-treated WT cells, cluster 4 was strongly enriched for pathways related to MAVS, type I and type II IFN signaling, and antiviral responses. Strikingly, the presence of these same signatures in NT *IRF1* KO cells suggests IRF1 to be a negative regulator of these pathways, suggesting that under steady-state conditions IRF1 also functions as a repressor of MAVS activation. Indeed, in Figure S1, *C* and *E*, we show that IRF7 and IRF9 abundance is increased upon *IRF1* KO, which may contribute toward the observed increased expression of other ISGs. In contrast, analysis of cluster 1, which constitutes gene transcripts with reduced abundance in *IRF1* KO cells, with or without IFN-γ treatment, revealed strong associations with pathways involved in the IL-6 response and STAT3 signaling, both of which are central to immune regulation. Additionally, we identified in cluster 1 enrichment of the Th1 pathway-related genes, which signal through IFN-γ and play a pivotal role in adaptive immunity (63, 64). These findings further underscore the multifaceted regulatory role of IRF1 in orchestrating both innate and adaptive immune responses.

Overall, our results highlight the critical function of IRF1 in fine-tuning IFN signaling pathways and maintaining immune homeostasis. They also offer a mechanistic explanation for the constitutive nuclear localization of IRF1, where it continuously associates with gene promoter elements, even in the absence of specific external stimuli (65).Through this ongoing engagement, IRF1 safeguards the balance of gene expression necessary for preserving cellular integrity and preventing aberrant activation of immune pathways.

### IRF1 OE confers antiviral protection through IFNAR-dependent but also JAK-independent pathways

**Figure 3:**
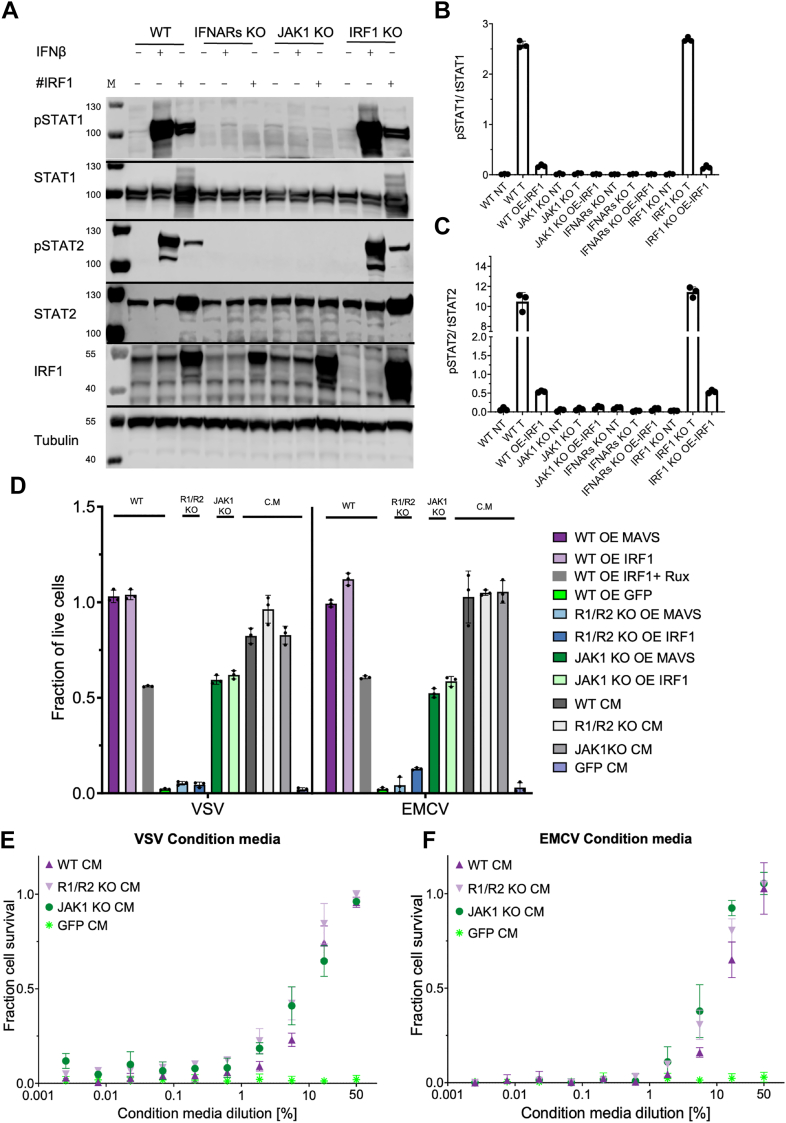
**Effects of IRF1 OE on STAT signaling and antiviral activity in different KO cell lines.***A*, STAT phosphorylation and IRF1 protein abundance in HeLa (WT), *IFNAR* KO, *JAK1* KO and *IRF1* KO cells after 30 min of treatment with 1 nM IFN-β, relative to nontreated cells. #IRF1 designate cells transiently transfected with *IRF1* for 48 h prior to treatment. *B*, normalized pSTAT1 abundance relative to total STAT1 abundance (three replicates). *C*, normalized pSTAT2 relative to total STAT2 abundance (three replicates). Quantification of IRF1 is presented in Figure S4*A*. *D*, HeLa WT, *IFNAR* KO (*R1/R2* KO), and *JAK1* KO cells were transiently transfected for 48 h with IRF1 (OE), MAVS (OE), or GFP (OE) (negative control). Where indicated, WT IRF1 OE cells were co-treated with the pan-JAK inhibitor ruxolitinib (IRF1+Rux). Additional conditions included treatment with conditioned media (CM) collected from WT, *IFNAR* KO (*R1/R2* KO CM), *JAK1* KO (JAK1 KO CM), or GFP-transfected (GFP CM) cells. Following transfection or CM treatment, cells were infected with VSV for 18 h or EMCV for 20 h. Cell viability was assessed by crystal violet staining and expressed as the fraction of live cells. Bars represent mean ± SD from three independent experiments. Statistical significance was determined using one-way ANOVA followed by Tukey’s multiple comparisons test. *E*, antiviral activity against VSV for WT HeLa. Cells were treated with CM from WT, *IFNAR* KO, and *JAK1* KO cells for 4 h before infection with the VSV for 18 h. Cells were stained with crystal violet for cell viability. *F*, antiviral activity against EMCV for WT HeLa. Cells were treated with CM from WT, *IFNAR* KO, or *JAK1* KO for 4 h before infection with the EMCV for 20 h. Cells were stained with crystal violet for cell viability. EMCV, encephalomyocarditis virus; IFNAR, IFN-α receptor; IFN, interferon; IRF1, interferon regulatory factor 1; KO, knockout; OE, overexpression; VSV, vesicular stomatitis virus.

To gain further insight into the role of IRF1 in regulating gene expression, we examined the effects of IRF1 OE through transient transfection. OE of IRF1 resulted in JAK1- and IFNAR-dependent phosphorylation of STAT proteins 48 h post-transfection (Figs. 3, *A*–*C* and S4*A*), suggesting that IRF1-mediated STAT activation requires an intact type I IFN signaling pathway. The activated STATs in IRF1 OE cells drove antiviral protection against VSV and EMCV even in the absence of IFN-β treatment (Fig. 3*D*). In contrast, no antiviral effect was detected in *IFNAR* KO cells, even when IRF1 was overexpressed, and IFN-β was added at saturating concentrations (Fig. S4*B*). Surprisingly, IRF1 OE in *JAK1* KO cells resulted in partial antiviral protection (Fig. 3*D*), despite lack of STAT1 or STAT2 phosphorylation (Fig. 3*A*), a phenomenon similar to that observed upon MAVS OE, which is known to induce IFN-β secretion (12). Further support for JAK-independent antiviral protection was gained by treatment with the pan-JAK inhibitor ruxolitinib (66); even with JAK inhibition, IRF1 OE elicited a partial antiviral response, similar to that observed in *JAK1* KO cells (Fig. 3*D*). To investigate whether antiviral protection was mediated by secreted molecules, we prepared conditioned media (CM) from four different cells: WT, *JAK1* KO, *IFNAR* KO, and GFP OE. CM were collected 48 h post-transfection (and thus OE) of either IRF1 or GFP (which serves a control for the effect of transient transfection), centrifuged, and filtered through a 0.22 μm syringe filter to remove cells and cellular debris and to ensure sterility. WT cells were subsequently incubated with the conditioned media, and antiviral activity was assessed. (Fig. 3*D*). To evaluate the efficacy of the CMs against VSV and EMCV, they were diluted, and antiviral protection was measured (Fig. 3, *E* and *F*). Except for the GFP CM, which served as a negative control, all other CMs provided full protection. Dilution of the CMs progressively reduced the antiviral activity, confirming that the protective effect was concentration-dependent and mediated by molecules present within the media.

These results indicate that IRF1 OE induces a robust antiviral response that mirrors the effects of type I IFNs, relying on intact type I IFN receptors in the recipient cells but not in the producer cells. Notably, partial antiviral activity was still observed in the absence of JAK1 both in *JAK1* KO cells and in ruxolitinib-treated cells suggesting that IRF1 can elicit a degree of antiviral protection through a JAK1-independent, paracrine mechanism. Obvious candidates here would be one of the noncanonical IFN induced pathways, including MAPK, PI3K/mTOR, and others (67). While U-STAT complexes where also shown to promote some degree of activity (41), this does not seem to be the case here, as STAT1 and STAT2 abundance were not increased in IRF1 OE in the background of *JAK1* KO (Fig. 3*A*).

### IRF1 OE induces secretion of type I IFNs and potent antiviral factors

**Figure 4:**
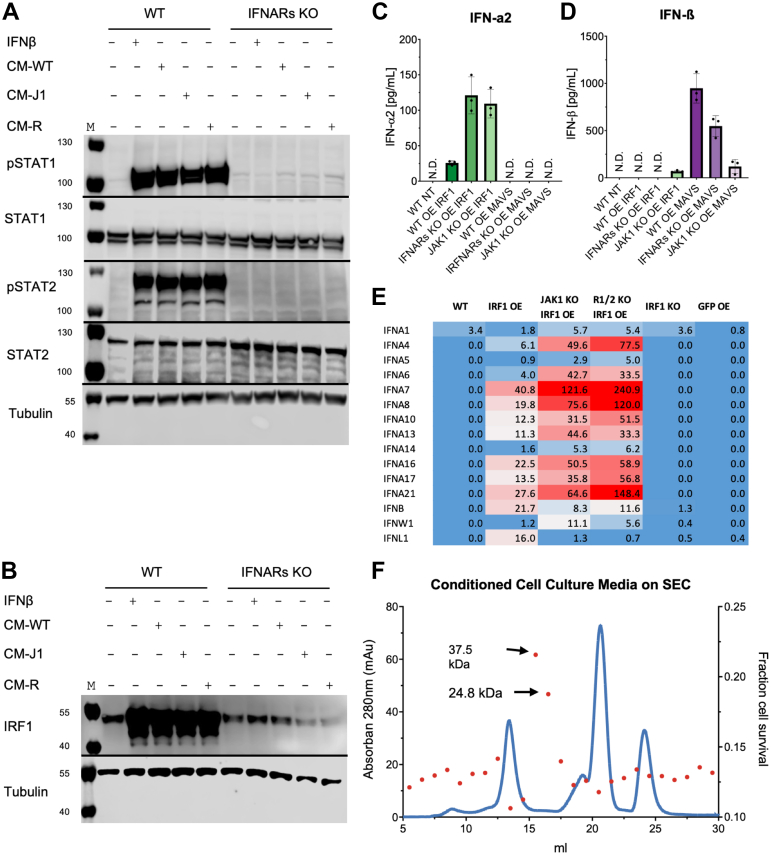
**Cytokine profiling and antiviral activity of conditioned media from IRF1 OE cells.***A*, STAT phosphorylation of HeLa cells (WT) and HeLa *IFNAR* KO after 30 min of treatment with CM from WT, J*AK1* KO (CM-J1), and *IFNAR* KO (CM-R) IRF1 OE cells, relative to nontreated cells. Quantification of this blot and two more replicates are presented in Figure S5, *A* and *B*. *B*, IRF1 abundance in HeLa (WT) and HeLa *IFNAR* KO cells following 6 h of treatment with CM from WT, *JAK1* KO, and *IFNAR* KO cells, relative to nontreated cells. Quantification of this blot is presented in Figure S5*C*. *C* and *D*, cytometry analysis of IFN-α2 (*C*) and IFN-β (*D*) abundance in cells overexpressing IRF1 (IRF1 OE) or MAVS (MAVS OE) in WT, *JAK1* KO, and *IFNAR* KO cells. WT NT cells were used as a negative control. *E*, heatmap of the normalized counts of various type I IFNs retrieved from the RNA-seq data. *F*, CM fractionation and their activity. The *blue line* represents absorption at 280 nm (mAu), while the *red dots* represent cell survival after treatment with the given fraction of CM for 4 h, followed by VSV infection for 18 h. Cell viability was determined by crystal violet staining. IFN, interferon; IFNAR, IFN-α receptor; KO, knockout; OE, overexpression; VSV, vesicular stomatitis virus.

The requirement of an intact IFNAR receptor suggests that IRF1 OE induces the secretion of type I IFNs into the medium. Previous studies have suggested a connection between IRF1 and the promoter of IFN-β (68). Our goal was to determine whether IRF1 promotes cytokine secretion and to identify the specific cytokines involved. To investigate this, we prepared CM as described above and measured STAT protein phosphorylation by WB. CM prepared from WT and *IFNAR* KO cells where IRF1 was overexpressed were added to WT and *IFNAR* KO cells for 30 min (Figs. 4*A* and S5, *A* and *B*). We observed robust STAT phosphorylation of WT cells for CM from WT and *IFNAR* KO cells, whereas no phosphorylation was detected upon adding CM to IFNAR KO cells, confirming the dependence on functional IFNAR signaling. In addition to analyzing STAT phosphorylation, we also examined the effect of CM on IRF1 protein abundance. After a 6-h induction with CM, we detected a marked increase in IRF1 protein abundance in WT cells (Figs. 4*B* and S5*C*). To identify which cytokines were present, we utilized the LEGENDplex Human Type 1/2/3 IFN Panel (69), which allows for the simultaneous quantification of multiple cytokines, including IFN-α, IFN-β, IFN-λ1, IFN-λ2/3, and IFN-γ (Figs. 4, *C* and *D*, and S5, *D* and *E*). In addition to IRF1, we tested MAVS OE, known to induce secretion of IFN-β and IFN-λ1 (70, 71). Our findings show high concentrations of IFN-β and IFN-λ1 in cells overexpressing MAVS, with lower concentrations being secreted from KO cells, suggesting a positive feedback loop. MAVS OE resulted in IFN-λ2/3 secretion only from WT cells. IRF1 OE mainly drove IFNα2 secretion, which was higher in *IFNAR* KO or *JAK1* KO cells, suggesting a negative feedback mechanism. A small amount of IFN-β was detected only in *JAK1* KO IRF1 OE cells. Next, we evaluated gene transcript abundance of the different type I IFN genes upon IRF1 OE and *IRF1* KO (Fig. 4*E*). RNAseq showed that the expression of many of the IFN-α subtypes were highly increased, even in the *JAK1* KO IRF1 OE and *IFNAR* KO IRF1 OE cells, providing further evidence of positive feedback of IRF1 activity through inducing type I IFN production, even in the absence of type I signaling. GFP OE, which is used as transfection control, did not show any increased gene expression, as was the case also for *IRF1* KO. Aiming to analyze the CM further, we prepared CM from both WT and *IFNAR* KO cells and after concentration loaded it onto a SEC column (Superdex 200 10/300 GL), collecting 1 ml fractions. Each fraction was tested for antiviral activity against VSV (Fig. 4*F*), identifying two active fractions at approximately 37.5 kDa and 24.8 kDa. Mass spectrometry analysis of these fractions revealed three unique peptides. Two peptides from IFN-α1: HDFGFPQEEFDGNQFQK and VGETPLMNADSILAVK (Figs. S6*A* and S7*A*) and one peptide from IFN-α4: DRHDFGFPEEEFDGHQFQK (Figs. S6*B* and S7*B*). The sequence similarity between IFN-α genes complicates the analysis for characterization of IFN-α subtypes. Together, these findings demonstrate that IRF1 OE induces the secretion of type I IFNs, particularly IFN-α subtypes, establishing a paracrine antiviral defense mechanism that amplifies the immune response.

### IRF1 orchestrates both interferon-dependent and -independent immune gene programs

**Figure 5:**
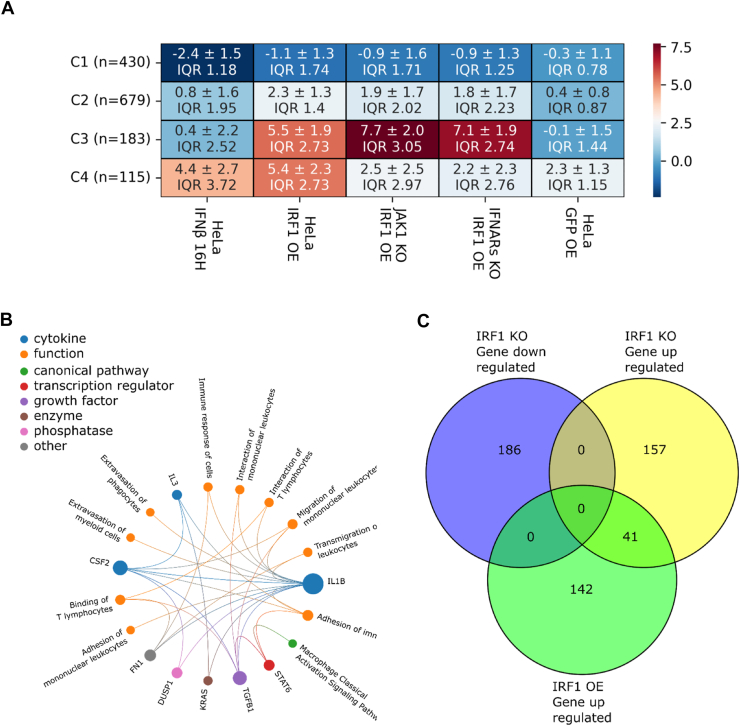
**Gene regulation and signaling pathway alterations upon IRF1 overexpression.***A*, heatmap showing hierarchical clustering of genes with significant expression changes (|log2(FC)| > 2) across WT IRF1 OE, *JAK1* KO IRF1 OE, and *IFNAR* KO IRF1 OE cells 48 h post-transfection, with WT cells treated with 2 nM IFN-β for 16 h and WT GFP OE used as control. Clusters are divided into four groups based on median expression. Standard deviation and IQR were calculated for each group. *B*, pathway analysis of Cluster 3 was performed using QIAGEN Ingenuity Pathway Analysis (IPA). Pathways were selected based on a z-score > 2 and a *p*-value of overlap < 0.05, indicating significant pathway enrichment in genes which expression is upregulated by IRF1 OE. *C*, Venn diagram comparing gene sets from different clusters, showing genes which expression is upregulated in *IRF1* KO cells (Cluster 4, Fig. 2) *versus* those upregulated in IRF1 OE cells (Cluster 3, Fig. 5A) and downregulated in *IRF1* KO cells (Cluster, 1 Fig. 2). IFNAR, IFN-α receptor; IRF1, interferon regulatory factor 1; KO, knockout; OE, overexpression.

To further dissect the molecular consequences of IRF1 OE and to identify the broader gene expression changes involved, we transfected IRF1 for 48 and extracted RNA from WT IRF1 OE, JAK1 KO IRF1 OE, and *IFNAR* KO IRF1 OE cells for RNA-seq analysis. Plotting log_2_(FC)| > 2.5 of IFN-β (Fig. S8*A*) or IFN-γ-treated cells (Fig. S8*B*) in relation to IRF1 OE cells shows that indeed gene transcript abundance upon IFN-β treatment mimics that observed upon IRF1 OE, while this is not the case for IFN-γ-treated cells. Next, we compared FC gene transcript abundance of IRF1 OE in *JAK1* KO background to *IFNAR* KO background cells relative to WT and found a tight correlation (Fig. S8*C*). However, relating FC of IRF1 OE to IRF1 OE in *IFNAR* KO cells shows large deviations between the two, further corroborating that IRF1 OE affects gene transcription directly and through a paracrine pathway (Fig. S8*D*). For a more detailed analysis, NT WT, *JAK1* KO, and *IFNAR* KO cells served as baseline controls for FC calculations. Differential expression analysis was performed using the UTAP pipeline (60) and DESeq2 package (61). Additionally, for comparison, we included WT cells treated with 1 nM IFN-β for 16 h to assess IFN-induced gene activation, and WT cells overexpressing GFP (WT GFP OE) as a negative control to account for any transfection-induced upregulation of type I IFN responses (62). All genes exhibiting significant expression changes relative to baseline (|log_2_(FC)| > 2) were extracted and organized into a heatmap. The heatmap was generated by hierarchical clustering and divided into four distinct groups based on expression patterns. For each cluster, the median expression and interquartile range (IQR) were calculated (Fig. 5*A*). The most notable expression patterns were observed in clusters 3 and 4, which exhibited distinct regulatory characteristics. Cluster 4 shows strong upregulation in both WT IRF1 OE cells and WT cells treated with IFN-β and was absent in *JAK1* KO and *IFNAR* KO cells (which slightly increased gene induction, similar to that observed in the GFP control). This support the interpretation that cluster 4 genes represent classical type I IFN-stimulated genes dependent on intact type I IFN signaling. In contrast, cluster 3 comprises of genes that were robustly upregulated in IRF1 OE cells and not dependent on IFN-β signaling or the presence of JAK1 or IFNAR. This indicates that their expression is independent of type I IFN signaling (for specific genes of cluster 3 see Fig. S8*E*). These findings suggest that IRF1 can initiate a distinct transcriptional program that bypasses the canonical IFN–JAK–STAT pathway. Pathway analysis of cluster 3 was performed using QIAGEN IPA (62). This analysis revealed significant enrichment of pathways related to the innate immune system, including genes such as IL1B, IL3, and the STAT6 signaling pathways (Fig. 5*B*). In addition, strong activation of pathways associated with the adaptive immune system were observed, including “interruption of T lymphocytes” and “adhesion of mononuclear leukocytes,” among others. Thus, beyond its role in inducing type I IFN secretion and upregulating ISGs, IRF1 OE can also directly activate genes involved in the innate and adaptive immune systems. Another interesting observation emerged from the Venn diagram analysis (Fig. 5*C*), where we compared the different clusters. We identified 142 distinct genes that were upregulated upon IRF1 OE and 157 genes upon *IRF1* KO. However, the abundance of 41 gene transcripts was increased both by IRF1 OE and *IRF1* KO, highlighting that IRF1 can act both as a transcriptional activator and a repressor on the same gene.

Collectively, these results demonstrate that IRF1 modulates immune gene expression through two parallel mechanisms: by driving interferon-dependent antiviral programs and by directly activating innate and adaptive immune pathways independently of canonical type I IFN signaling.

### Characterization of IRF1 DNA-binding specificity using protein binding microarrays

**Figure 6:**
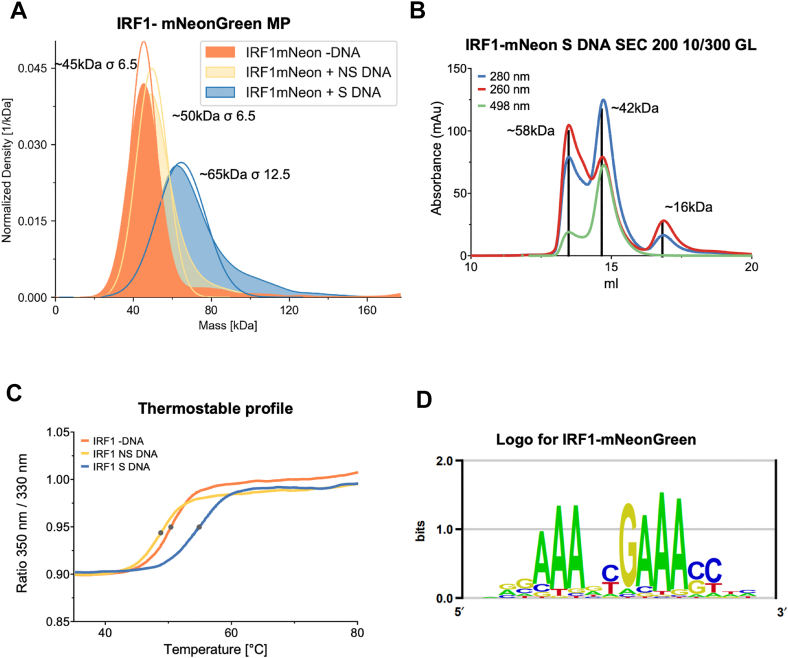
**Verification of IRF1 DNA-binding specificity using mass photometry and size-exclusion chromatography.***A*, mass photometry of the IRF1-mNeonGreen without DNA (-DNA) with scramble DNA (+NS) and with specific DNA (+S DNA). The data shown here represent one example from three independent biological replicates. *B*, size-exclusion chromatography of IRF1 mNeon incubate for 4 h with specific DNA. The *blue line* shows absorbance at 280 nm, the *red line* 260 nm, and the *green line* 498 nm. *C*, thermal stability of IRF1-mNeonGreen without DNA (-DNA) with scramble DNA (+NS) and with a specific DNA (+S DNA). *D*, the energy-normalized binding motif logo for IRF1-mNeonGreen was generated using the enoLOGOS software (74). IRF1, interferon regulatory factor 1.

We hypothesized that the distinct regulatory effects of IRF1 on transcription as observed upon its KO or OE may arise from variations in its DNA-binding affinity to different promoter regions. To explore this possibility, we aimed to determine IRF1 binding to promoter regions of type I IFN-induced genes and genes differentially regulated by IRF1 OE and KO. At first, we verified the DNA-binding motif of IRF1 by performing PBM experiments (72, 73). For this, we purified the IRF1 DBD, comprising amino acids 1 to 136 (MW16 kDa). To enable fluorescence-based detection, the DBD was conjugated to mNeonGreen, a fluorescent protein with a MW of 26 kDa, resulting in a fusion protein of 42 kDa. The fusion protein was expressed in *E. coli* and purified by cation exchange chromatography using SP Sepharose, taking the advantage of the high isoelectric point (pI = 10.2) of the DBD for direct column binding, followed by elution with high-salt buffer (Fig. S9*A*). The protein was further purified by SEC using a Superdex 75 PG column (Fig. S9*B*). Following purification, we evaluated the DNA-binding specificity of the fusion protein. Two double-stranded DNA oligonucleotides were synthesized: one containing a previously reported IRF1 consensus binding sequence (5′-GAGAAGTGAAAGTACTTTCACTTCTC-3′) (54) and a scrambled control sequence with identical nucleotide composition (5′-ATATTACGTCGCACTAGGATAGATCT-3′). Each oligonucleotide had an approximate MW of 16 kDa. Mass photometry analysis was conducted on three samples: IRF1 DBD-mNeonGreen without DNA, with nonspecific (scrambled) DNA, and with specific target DNA (Fig. 6*A*). The IRF1 fusion protein alone exhibited a mass of approximately 45 kDa. Adding scrambled DNA produced a slight increase to ∼50 kDa (likely reflecting minor nonspecific interactions), and adding specific target DNA resulted in a mass of ∼65 kDa. This confirmed the specific and stable binding of IRF1 DBD to its target DNA sequence, despite the highly positive charge of the DBD. Subsequent size measurements were conducted by SEC on a Superdex 200 10/300 Gl column. Three distinct wavelengths were monitored: 280 nm, corresponding primarily to protein absorbance; 260 nm, indicative of nucleic acid (DNA) absorbance; and 498 nm, corresponding to the fluorescence emitted by the mNeonGreen tag conjugated to the IRF1 DBD. The IRF1 DBD-mNeonGreen fusion protein was incubated with the specific DNA sequence for 4 h prior to SEC analysis (Fig. 6*B*). Parallel measurements were performed using scrambled DNA and in the absence of DNA as controls (Fig. S10, *A*–*C*). SEC analysis of the specific DNA-bound sample revealed a major peak corresponding to an apparent molecular mass of ∼58 kDa, with a dominant 260 nm signal relative to 280 nm, indicative of the fusion protein bound to DNA. A second prominent peak was detected at ∼42 kDa, with a higher 280 nm signal, corresponding to unbound IRF1 DBD-mNeonGreen. A third peak at ∼16 kDa, characterized by a dominant 260 nm signal, corresponds to free DNA oligonucleotides. Next, we assessed the thermal stability of the IRF1 fusion protein under different binding conditions: without DNA, with scrambled DNA, and with specific DNA. As shown in Figure 6*C*, binding to the specific DNA sequence resulted in a higher melting temperature, consistent with the increased stability typically observed in protein–DNA complexes compared to the unbound protein. Having confirmed the functionality and stability of the IRF1 DBD-mNeonGreen fusion protein, we proceeded with the PBM experiment to characterize the preferences of IRF1-DBM to bind DNA. The PBM analysis successfully identified a sequence-specific binding motif, which was visualized as an energy-normalized sequence logo using the enoLOGOS software (74) (Fig. 6*D*). These results confirmed the sequence specificity of IRF1 DNA binding and provided a foundation for predictive modeling of IRF1 occupancy across promoter regions of interferon-regulated genes.

### Predictive modeling of IRF1 promoter occupancy reveals functional binding sites in antiviral gene networks

**Figure 7:**
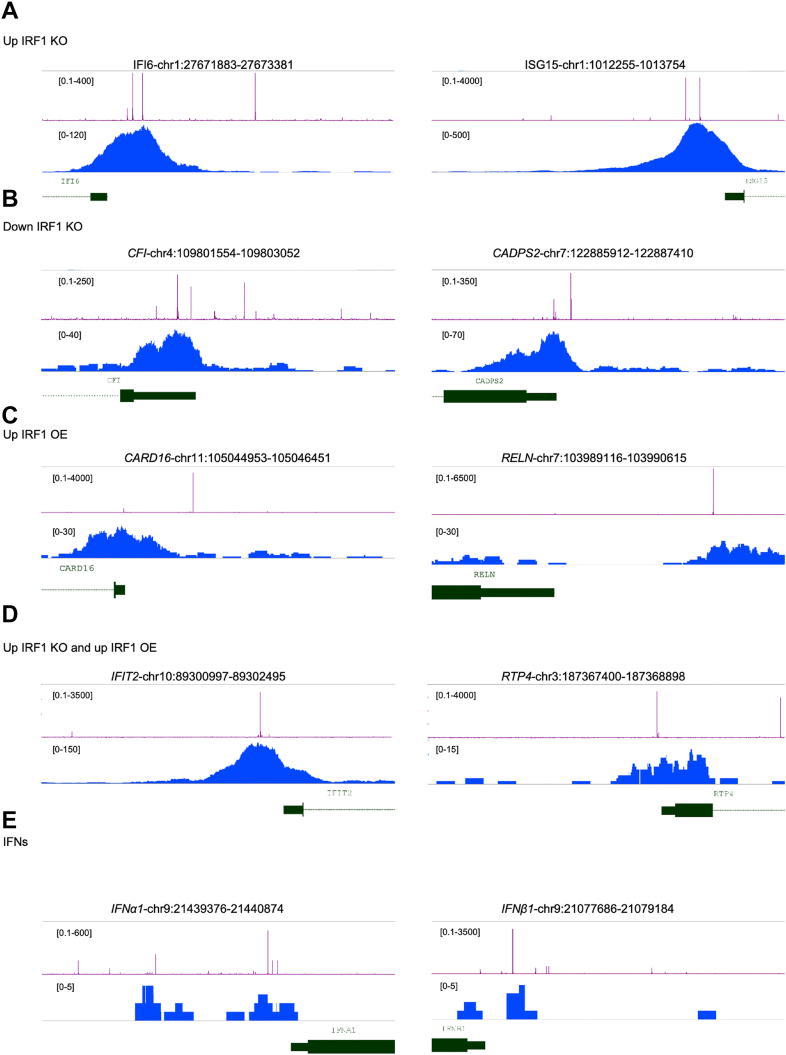
**Predicted and observed IRF1 binding in promoter regions of key immune-related genes.***A–E*, comparison of predicted IRF1-binding affinity and ChIP-seq coverage in promoter regions of genes identified from RNA-seq analysis. The promoter regions include 1000 base pairs upstream and 500 base pairs downstream of the transcription start site. Predicted IRF1-binding affinity is shown in *purple* and was visualized using inverse log_2_-transformed z-scores (2^∧^z-score) to represent the relative strength of predicted interactions on a linear scale. Raw ChIP-seq data were reprocessed with a standardized pipeline from GEO datasets (GSM6928615, GSM6928616) represented in *blue*, showing IRF1 binding coverage across the promoter regions. Gene annotations are in *green*. *A*, gene transcripts showing upregulation in the *IRF1* KO, corresponding to cluster 4 from RNA-seq analysis (Fig. 2*C*). *B*, gene transcripts showing downregulation in the *IRF1* KO, corresponding to cluster 1 from RNA-seq analysis (Fig. 2*C*). *C*, gene transcripts showing upregulation in IRF1 OE, corresponding to cluster 3 from RNA-seq analysis (Fig. 5*A*). *D*, gene transcripts showing upregulation in both the *IRF1* KO and IRF1 OE (Fig. 5*C*). *E*, analysis of the IFN promoter region, where a subtle ChIP-seq peak aligns with predicted binding sites. Although this peak is less pronounced, it may be functionally significant due to the high sensitivity of IFN receptors, which can initiate an antiviral response even with very low-level of type I IFN. More genes are shown in Figure S12. GEO, Gene Expression Omnibus; IFN, interferon; IRF1, interferon regulatory factor 1; KO, knockout; OE, overexpression.

To further investigate IRF1-binding dynamics, we developed a predictive model based on binding affinities derived from DNA sequence data. Using the output of our PBM analysis, we first generated a sequence logo and calculated binding affinities for all possible 7-mer sequences, representing potential IRF1 recognition motifs. We then analyzed the promoter regions of genes identified through our RNA-seq clustering analysis, focusing on three key groups: 1) Genes which transcript abundance was reduced in cluster 1 in *IRF1* KO cells, representing genes were IRF1 is a transcription factor independent on IFN (Fig. 2*C*); 2) genes which transcript abundance increased upon *IRF1* KO (cluster 4), representing genes which expression is repressed by IRF1, but induced by IFN. 3) Genes which transcript abundance is increased upon IRF1 OE (cluster 3, Fig. 5*A*), corresponding to genes were IRF1 is an inducer of transcription, independent on IFN. For each gene of interest, we extracted its promoter region defined as 1000 base pairs upstream and 500 base pairs downstream of the transcription start site, adjusted for gene orientation. Using the PBM-derived binding affinities, we constructed a gene-specific model predicting IRF1 binding strength and location across each promoter region (Fig. S11). This model allowed us to identify high-confidence candidate binding sites for the IRF1 protein. To validate the model's predictive power, we re-analyzed IRF1 ChIP-seq data from the Gene Expression Omnibus (GEO) database (GSM6928615, GSM6928616) (43), which originated from a previous study examining time-dependent recruitment of GAS, ISGF3, and IRF1 complexes to GAS, ISRE, and composite elements. Data were processed using the Galaxy platform (75) and visualized with the integrated genome browser (IGB) (76) (Figs. 7 and S12). Although the original ChIP-seq was performed in Huh7.5 cells, we adapted the analysis to HeLa cells to align with our experimental system. Remarkably, our analysis revealed strong ChIP-seq peaks that overlapped with high-affinity binding sites predicted by our model (Figs. 7, *A*–*D*, and S12, *A*–*D*), supporting the model's accuracy in identifying *bona fide* IRF1 regulatory elements.

Notably, while the type I IFN promoter regions lacked a dominant ChIP-seq peak in the original analysis, our model predicted high-affinity binding sites (Figs. 7*E*, S11*E*, S12*E*). Closer inspection revealed a small but distinct ChIP-seq signal aligning with the predicted sites an observation that is biologically meaningful given that even minimal amounts of IFN can trigger a potent antiviral response due to the picomolar concentrations needed to activate a biological response (77, 78). Thus, weak DNA binding may be sufficient to initiate a biologically relevant response, supporting the value of our model in identifying subtle yet functional regulatory elements.

These results show that while IRF1 consensus motif (GAAANNGAAA) has been known for decades (28, 38, 53), our PBM-derived energy-normalized matrices reveal graded affinity differences for all 7-mer variants, offering predictive power far beyond motif presence or absence. This allows for quantitative ranking of promoter binding potential, which classical motif searches or position weight matrices cannot achieve. Overall, our findings demonstrate that IRF1 binding is highly sequence specific and that its promoter occupancy can be accurately predicted using a binding affinity-based model. By integrating these predictions with re-analyzed ChIP-seq data, we identified both prominent and subtle IRF1-binding events across key immune gene promoters, including type I IFNs particularly IFN-α subtypes and canonical ISGs. These results establish IRF1 as a central transcriptional regulator of antiviral immunity, capable of directly modulating both IFN-α expression and downstream immune gene networks. This provides a mechanistic framework for understanding the distinct regulatory effects of IRF1 under KO and OE conditions.

### Targeted promoter analysis of IFIT2 reveals functionally predictive IRF1-binding sites

**Figure 8:**
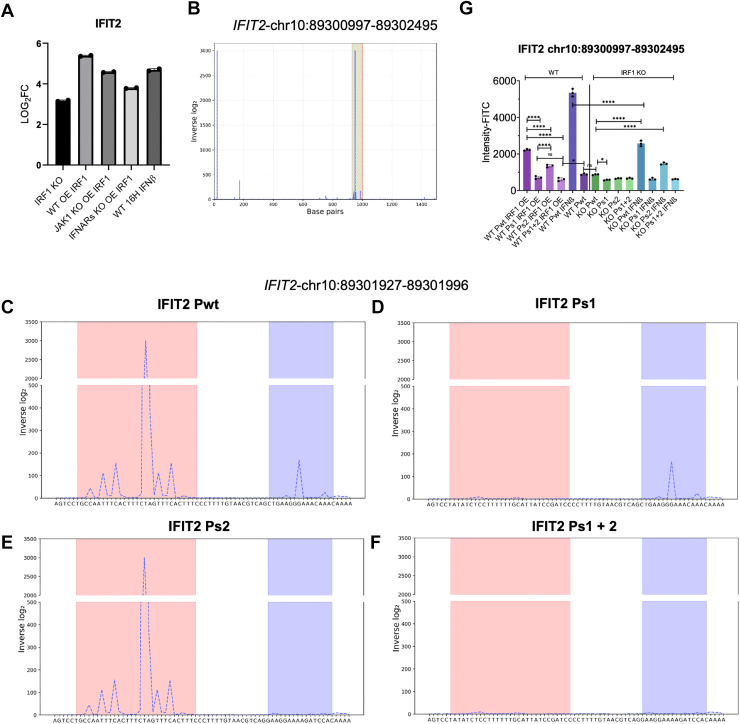
**Functional analysis of IRF1 regulation of the IFIT2 promoter using prediction, mutagenesis, expression assays, and large-scale transcriptomic correlations.***A*, normalized RNA-seq expression of IFIT2 in HeLa cells under the indicated conditions: *IRF1* KO, IRF1 OE, *JAK1* KO + IRF1 OE, *IFNAR* KO + IRF1 OE, and WT treated with IFN-β for 16 h. *B*, predicted IRF1 binding affinity across the IFIT2 promoter (chr10:89,300,997–89302495), based on inverse log_2_-transformed z-scores. The analyzed region includes 1000 bp upstream and 500 bp downstream of the transcription start site. Two predicted binding sites near the start codon are highlighted in *green*. *C–F*, predicted IRF1 binding sites on the WT IFIT2 promoter (*C*), and three scrambled variants in which one or both predicted IRF1 binding sites were scrambled: Ps 1 (*D*), Ps 2 (*E*), and Ps 1 + 2 (*F*). Binding affinity was recalculated for each sequence and plotted using inverse log_2_-transformed z-scores. *G*, reporter assay results for the IFIT2 promoter fused with the eUnaG2 gene expressed in WT and *IRF1* KO HeLa cells. Cells were transfected with reporter constructs containing the WT promoter or scrambled variants and were either NT, transfected with IRF1 OE, or treated with IFN-β for 12 h. Reporter expression is shown as fold change in fluorescence intensity measured by flow cytometry. Data represent mean ± SD from independent experiments, from which significance was calculated using one-way Anova with post Tukey test. *P* values of 0.05, and <0.001 are shown as ∗ and ∗∗∗∗. IFIT2, tetratricopeptide repeats 2; IFN, interferon; IFNAR, IFN-α receptor; IRF1, interferon regulatory factor 1; KO, knockout; Ps1, first, higher peak scrambled; Ps2, second (lower) peak scrambled; Ps 1 + 2, both sites scrambled; NT, untreated; OE, overexpression.

To experimentally validate our predictive binding model, we selected IFIT2 as a representative target gene for detailed analysis. IFIT2 is a well-characterized type I IFN ISG, which expression is rapidly induced following IFN-α/β signaling. Its role in antiviral defense is modulating cellular responses to viral RNA, with its transcription being tightly regulated at the promoter level (79, 80, 81). We chose IFIT2 because it exhibited consistent upregulation under multiple conditions: in *IRF1* KO cells, in IRF1 OE cells, and following IFN-β treatment. Notably, increased IFIT2 expression was also observed in both *JAK1* KO IRF1 OE and *IFNAR*s KO IRF1 OE cells (Fig. 8*A*, log_2_-fold change from RNA-seq), highlighting its robust transcriptional activation across distinct genetic backgrounds and signaling contexts. Using our IRF1 promoter binding model, we analyzed the IFIT2 promoter region (chr10:89300997–89302495), defined as 1000 base pairs upstream and 500 base pairs downstream of the transcription start site. The model identified two high-affinity IRF1 binding peaks near the start codon, which is marked in green in Figure 8*B*. A focused view of this region (chr10:89301927–89301996, ∼70 bp) is shown in Figure 8*C* (WT), highlighting the location of the two predicted binding sites.

To evaluate the contribution of each predicted site, we generated three scrambled versions of the sequence: one with the first, higher peak scrambled (Ps1, Fig. 8*D*), one with the second (lower) peak scrambled (Ps2, Fig. 8*E*), and one with both sites scrambled (Ps 1 + 2, Fig. 8*F*). The predictive model was re-run on each of the modified sequences, alongside the wildtype control. As shown in Figure 8, *C*–*F*, scrambling of either site reduced the predicted IRF1-binding potential, and scrambling both eliminated it entirely. Notably, the first peak exhibited a higher predicted binding affinity than the second, and we therefore hypothesized that its disruption (Ps1) would have a more pronounced effect on promoter activity than disruption of the second peak. To test this hypothesis, we synthesized (by GenScript) a 600 bp fragment of the IFIT2 promoter (chr10:89300997–89302495) and cloned it upstream of an eUnaG2 fluorescent reporter. This construct allowed us to assess promoter activity under various experimental conditions in life HeLa cells. WT and *IRF1* KO cells were transfected with wildtype and scrambled promoter-reporter constructs. Forty-eight hours post-transfection, fluorescence intensity was measured by flow cytometry. The experiment was conducted under three conditions: NT, IRF1 OE, and IFN-β treatment for 12 h. All conditions were tested for the four constructs: WT, Ps 1, Ps 2, and Ps 1 + 2. Transfecting WT HeLa cells with the WT promoter construct showed significantly increased fluorescence either upon IRF1 OE or IFN-β treatment. This is consistent with increased transcription activity. In contrast, all three scrambled constructs exhibited reduced fluorescence intensity under these conditions, indicating disruption of IRF1-mediated promoter activation. Among the scrambled constructs, Ps 2 retained the highest activity, while Ps 1 and Ps 1 + 2 showed markedly reduced signal, aligning with our prediction that the first binding site is more important. In *IRF1* KO cells, baseline fluorescence remained low across all constructs, and no induction was observed in the absence of stimulation. However, upon IFN-β treatment, the wildtype promoter again showed increased fluorescence, whereas the Ps 1 and Ps 1 + 2 variants remained less responsive. Analyzing gene transcript abundance of IFIT2 against multiple IRFs across 1206 human cell types from the Human Protein Atlas (58), shows a positive association between IRF1 and IFIT2 expression, consistent with our promoter-based findings that IRF1 directly activates IFIT2 transcription (Fig. 2*B*). This further supports the conclusion that IRF1 binding is required for full promoter activation and that the predicted motifs are functionally important.

Together, these findings confirm that IRF1 directly regulates the IFIT2 promoter through specific sequence elements and that our computational model reliably predicts functional IRF1-binding sites. By combining promoter mutagenesis, transcriptional reporter assays, and integrative modeling, we demonstrate that IRF1 binding is both necessary and sufficient for transcriptional activation of this key ISG under multiple signaling conditions. Furthermore, transcriptomic correlation across diverse human cell types reveals that the IRF1-IFIT2 relationship is broadly conserved, reinforcing the physiological relevance of our findings. This approach not only validates our motif-based prediction strategy but also reinforces the central role of IRF1 as a versatile regulator of antiviral gene expression.

## Discussion

Our investigation into the role of IRF1 in regulating interferon responses has revealed its multifaceted and context-dependent functions in both basal and induced immune states. Traditionally viewed as an ISG, IRF1 is often characterized primarily by its role in amplifying responses to IFN stimulation. However, our findings challenge this restricted view, showing that IRF1 also plays a constitutive role in regulating immune homeostasis and antiviral readiness, even in the absence of cytokine stimulation. Contrary to our initial hypothesis (11), *IRF1* KO did not impair the induction of ISGs upon IFN-β stimulation (Fig. S1*A*), suggesting that the canonical JAK-STAT pathway can compensate for the absence of IRF1 under acute type I IFN signaling. Conversely, gene transcription levels were significantly reduced in *IRF1* KO cells upon IFN-γ treatment. This is consistent with reports showing that ISG induction is mediated by STAT1, STAT2, and IRF9-containing complexes, which bind ISRE motifs and contribute to redundant or overlapping regulatory networks (82, 83, 84). On the other hand, GAS-containing promotors are dominantly affected by IRF1, as shown previously (11). Therefore, the nuanced role of IRF1 in this process may reflect this functional overlap in immune responses (43). In line with this hypothesis, our RNA-seq data and WB analysis revealed increased mRNA and protein abundance of other IRFs, specifically IRF3, IRF7, and IRF9, in *IRF1* KO cells (Fig. S1, *C* and *E*), suggesting potential compensatory transcriptional activity. Indeed, the IFIT2 promoter was activated both in the presence of IRF1 and upon its KO, to high levels (Fig. 8*G*).

To assess whether the regulatory relationships identified in HeLa cells extend to other human cell types, we leveraged transcriptomic data from 1206 cell types available through the Human Protein Atlas. Violin plot analysis of ISG abundance across these cell types revealed that expression in HeLa cells consistently lies near the median and that IRF1 abundance in different cell types is 100-fold higher or lower than in HeLa cells. (Fig. S2*A*). The observed variability in IRF1 abundance is in line with previous work, showing large cell-specific variations in the type I IFN responses (59), giving further weight to this study, where we compared the cellular responses of *IRF1* KO and OE to that observed in WT HeLa cells. We further examined the correlation between IRF1 and multiple immune genes, including OAS2, IFIT1, and IFIT2, and found consistent positive associations across the cell atlas (Figs. 2, *A* and *B*, and S2*B*). These relationships extended to other IRFs as well: IRF3, IRF7, and IRF9 expression were also positively correlated with ISG abundance. In particular, IRF9, which is upregulated in *IRF1* KO cells, displayed strong predictive power for ISG expression across tissues, consistent with a compensatory mechanism. Collectively, these findings highlight a broader regulatory logic wherein multiple IRFs contribute to ISG expression in overlapping yet context-specific patterns and underscore the relevance of our findings beyond the HeLa system.

Transcriptomic analysis revealed that IRF1 is critical for maintaining basal ISG expression under homeostatic conditions. *IRF1* KO cells exhibited substantial upregulation of antiviral genes such as *MX1*, *IFIT3*, and *IL6*, along with the downregulation of regulatory genes such as *AQP3* and *LPL* indicating a loss of transcriptional control that could prime cells for aberrant inflammatory responses (Fig. S1*B*) (85). Our pathway analysis using QIAGEN IPA revealed that IRF1 exerts a broad regulatory influence on immune signaling networks, functioning both as a transcriptional activator and repressor depending on gene context and signaling conditions. Without external stimulus, IRF1 suppresses key components of the innate immune system, including MAVS and type I/II interferon signaling pathways (Fig. 2*D*), while positively regulating other immune programs such as IL6/STAT3 signaling, which were downregulated in *IRF1* KO cells (Fig. 2*E*). These observations support a dual regulatory role for IRF1 in fine-tuning immune responses. Moreover, they are consistent with previous reports describing IRF1’s essential role in maintaining immune homeostasis, promoting basal gene expression, and preventing the aberrant activation of inflammatory and stress-related pathways (86, 87).

IRF1 OE triggered potent antiviral protection even in the absence of exogenous IFN treatment, indicating that IRF1 can activate an antiviral program autonomously. Indeed, RNA-seq showed IRF1 OE to induce gene transcription, which is partially similar to that observed upon IFN-β treatment (but not IFN-γ treatment, Figs. 5*A*, S8, *A*–*D*). This protection was strictly dependent on the presence of functional IFNARs in the recipient cells but was independent of both IFNARs and JAK1 in the IRF1-OE producer cells (Fig. 3*D*), suggesting that IRF1 can induce the secretion of antiviral factors that act in a paracrine manner. Notably, some antiviral protection persisted in *JAK1* KO IRF1 OE cells, as well as in cells treated with the pan-JAK inhibitor ruxolitinib. This observation suggests that IRF1 can mediate antiviral responses through alternative signaling routes independent of classical JAK-STAT activation, potentially involving noncanonical pathways such as MAPK or PI3K/mTOR signaling. These findings are consistent with previous reports showing that IRF1 can directly upregulate antiviral effectors without requiring JAK1-mediated STAT phosphorylation, supporting the idea of a noncanonical IRF1-driven antiviral pathway (84, 88).

While previous studies have shown that IRF1 OE can broadly induce antiviral responses (18), it was suggested that the antiviral response is not due to paracrine type I IFN signaling driven by IRF1 OE, and it was indeed shown that the response occurred also in *STAT* KO cells. Conversely, we propose that IRF1 OE activates two complementary mechanisms: one in which IRF1 OE drives the transcription of antiviral effectors directly within the same cell, and a second one in which IRF1 induces the secretion of type I IFNs that act in a paracrine manner on neighboring cells. The use of *IFNAR1/2* KO cells allowed us to functionally separate these two mechanisms: antiviral protection in recipient cells was entirely dependent on functional IFNAR signaling, whereas antiviral activity was still observed in IRF1-OE cells that are deficient in *JAK1* (Figs. 3*D* and S4*B*). Thus, the induced antiviral activity can also act through alternative, non JAK/STAT signaling, explaining the discrepancy between our data and previous reports (18). Importantly, our study also reveals an unexpected antiviral phenotype with enhanced resistance to VSV and EMCV infection in *IRF1* KO cells that were not treated with IFN (Fig. 1*E*). This phenotype likely arises from compensatory upregulation of other IRF family members, such as IRF9 and IRF7, which we validate at both the transcript and protein level (Figs. S1, *C*–*E* and S13, *A*–*C*). Thus, our findings uncover both canonical and compensatory antiviral programs regulated by IRF1 in a context-dependent manner.

Transcriptomic analysis of IRF1 OE cells revealed robust upregulation of multiple type I IFN genes, particularly members of the IFN-α family, including IFNA1, IFNA2, and IFNA4 (Fig. 4*E*). This upregulation was also detected in *JAK1* KO and *IFNAR* KO backgrounds, indicating that while feedback suppression mechanisms may exist, they do not fully inhibit IRF1-mediated transcription. Despite the increased abundance of some ISGs also in *IRF1* KO cells, no increased abundance of type I IFN gene expression was observed. At the protein level, mass spectrometry of concentrated conditioned media confirmed the presence of secreted IFN-α peptides, identifying two unique peptides corresponding to IFN-α1 and one corresponding to IFN-α4 (Figs. S6, *A* and *B*, and S7, *A* and *B*). These results support the hypothesis that IRF1 directly induces the secretion of specific type I IFN-α subtypes (89, 90, 91), which to our knowledge is the first direct evidence of IFN-α1 and IFN-α4 peptide secretion following IRF1 OE as confirmed by mass spectrometry. Furthermore, the LEGENDplex assay enables simultaneous detection of multiple interferons including IFN-α2, IFN-β, IFN-γ, IFN-λ1, and IFN-λ2/3 with high specificity and sensitivity. Using this assay, we directly detected IFN-α2 protein in the conditioned media of IRF1-overexpressing cells (Figs. 4, *C* and *D*, S5, *D* and *E*), confirming IRF1-induced cytokine secretion at the protein level and validating our transcriptomic and mass spectrometry findings.

Another interesting observation emerging from the transcriptomic analysis of IRF1-OE cells was the identification of a cluster of genes (C3) that were upregulated following IRF1 OE but not in response to IFN-β treatment (Figs. 5*A*, and S8*E*). These genes are part of the adaptive immune system, particularly those involving T cell signaling and function (Fig. 5*B*). This finding aligns with previous studies linking IRF1 to adaptive immunity, including its role in T cell development and maturation (47, 48). These results suggest that IRF1 may extend its regulatory influence beyond antiviral and innate immune responses, contributing to the modulation of adaptive immune programs under specific conditions.

In addition to its role in activating immune-related genes, our findings also highlight IRF1’s ability to fine-tune gene expression by acting as both a transcriptional activator and repressor. RNA-seq analysis of *IRF1* KO and OE revealed, in addition to distinct sets of upregulated and downregulated genes also a subset of genes that were upregulated in both *IRF1* KO and OE (Fig. 5*C*). This overlap suggests that IRF1 may exert opposing regulatory effects on certain genes depending on context potentially through secondary signaling effects or dosage-sensitive promoter interactions (89, 92, 93, 94). Our data suggest that this may be due to the increased abundance of IRF3, IRF7, and IRF9 in *IRF1* KO cells. Interestingly, IRF3 protein levels were elevated in *IRF1* KO cells regardless of treatment, suggesting a constitutive upregulation. IRF7 abundance increased in WT cells after IFN-β stimulation, as anticipated. However, its abundance increased also in NT *IRF1* KO cells. A similar trend was observed for IRF9. These results were corroborated by qPCR analysis, which mirrored the protein-level changes (Fig. S1, *C* and *E*). IRF1, IRF3, and IRF7 can bind ISRE-like sequences. However, IRF1 also recognizes single IRF-E half-sites (GAAA), whereas IRF7 typically binds dimeric GAAA sites. Prior studies have shown that IRF7 OE is sufficient to drive ISG induction and confer antiviral protection (95). Moreover, the highly increased abundance of IRF9 can also stimulate some ISG gene expression without type I IFN stimulation (96). Thus, the partial redundancy between the different IRFs can explain why some ISGs are activated in *IRF1* KO cells. Our data support a model in which IRF1 suppresses other IRFs under homeostatic conditions directly or indirectly. In its absence, IRF7, IRF3, and IRF9 become derepressed, contributing to the elevated ISG expression and partial antiviral resistance observed in *IRF1* KO cells.

PBM experiments were used to identify specific DBMs for IRF1, which were used to establish a predictive binding affinity model (Figs. 7, S11, and S12). Although our study did not directly assess chromatin accessibility using ATAC-seq or histone modification profiles as has been done previously (55, 56), we observed a substantial correlation between our predictive binding model and reanalyzed ChIP-seq data (Figs. 7 and S12). This supports the biological relevance of the predicted high-affinity IRF1 sites, including subtle peaks missed in the original ChIP-seq publication (43). While the IRF1-binding motif has been previously described (28, 38, 53), our PBM approach goes significantly beyond known consensus sequences. Instead of relying on simplified consensus motifs, our method yields full binding affinity matrices for all 7-mers, enabling quantitative predictions of differential binding across promoter landscapes. This energy-normalized model allows not only for prediction of discrete binding sites but also for ranking and evaluating binding strength, contributing a resolution previously unattainable through classical motif-based or ChIP-seq-only approaches. Incorporating chromatin context into future studies would further refine these predictions, but our findings already illustrate that PBM-derived affinity models, when combined with reanalyzed ChIP-seq, can reliably uncover functionally relevant promoter interactions. A particularly notable finding was the identification of a small but discernible peak in the IFN promoter region. Although this peak was initially underrepresented in the ChIP-seq data, our affinity-based model highlighted it as a potential binding site. This subtle signal is consistent with the biological context, as IFN receptors exhibit high sensitivity for their ligands. Furthermore, extending our promoter analysis to IRF3, IRF7, and IRF9, which are upregulated upon *IRF1* KO (Fig. S1*C*) showed strong predicted IRF1-binding affinity as well as ChIP-seq enrichment at the promoter region (Fig. S13, *A*–*C*), suggesting that IRF1 may directly regulate their transcription. These observations underscore the capacity of our model to detect binding events that, while subtle, play a crucial role in initiating immune responses. We validated our binding predictions using the IFIT2 promoter as a case study. This gene expression was upregulated in both *IRF1* KO and OE cells and served as a clean model to dissect promoter dynamics. By scrambling predicted IRF1-binding motifs and measuring eUnaG2 reporter expression, we showed that mutation of either binding site impaired promoter activity with the first site having the strongest predicted and functional impact (Fig. 8). These results demonstrate that IRF1 binding is both necessary and sufficient for driving transcription from target promoters and that our computational framework can successfully identify biologically relevant regulatory elements.

In conclusion, IRF1 emerges as a multifaceted regulator of immune responses, playing essential roles in both immune homeostasis and antiviral defense. Our study demonstrates that IRF1 not only orchestrates the secretion of key cytokines particularly type I IFNs but also fine-tunes the expression of immune-related genes through a dual function as both a transcriptional activator and repressor. This regulatory versatility allows IRF1 to maintain basal expression of ISGs and coordinate antiviral defenses, even in the absence of canonical JAK-STAT signaling. Importantly, the partial antiviral protection observed in *JAK1* KO cells highlights IRF1’s capacity to engage noncanonical signaling pathways potentially involving MAPK or PI3K/mTOR cascades thereby extending its functional reach beyond traditional IFN responses. These findings are particularly relevant in the context of cancer immunotherapy, where defects in JAK1 signaling can limit the efficacy of cytokine-based treatments (97, 98, 99). By driving innate immune programs independently of JAK-STAT activation, IRF1 represents a promising therapeutic target for enhancing antiviral and antitumor immunity in settings of signaling impairment. The successful application of our affinity-based predictive model further underscores the value of integrating computational and experimental approaches. This strategy enabled the identification of both dominant and subtle IRF1 binding events that may be overlooked by traditional ChIP-seq analysis, providing a more nuanced understanding of IRF1’s regulatory landscape.

Future studies should investigate how IRF1 cooperates with other transcription factors such as IRF3, IRF7, IRF9, STAT1, and STAT2 to coordinate gene expression networks and whether IRF1 binding affinity or chromatin context governs its context-specific activity. The ability of IRF1 to selectively induce IFN-α subtypes also opens exciting therapeutic avenues, particularly in immuno-oncology, where restoring or mimicking IRF1 function may reinstate immune competence in tumors with impaired upstream pathways.

## Experimental procedures

### Cell lines

HeLa cells, derived from a human cervical cancer cell line, were used for all experiments in this study and were cultured in Dulbecco's Modified Eagle's Medium (Gibco 41,965–039) supplemented with 10% fetal bovine serum (Gibco 12,657–029), 1% pyruvate (Biological Industries 03–042-1B), and 1% penicillin–streptomycin (Biological Industries 03–031-1B).

### Generation of KO cells with CRISPR-Cas9

All KO cell lines in this study were generated with CRISPR-Cas9 technology, as previously described (11, 100). For the current study, we generated *IRF1* KO HeLa cell lines. To target the appropriate genes, we designed a single guide RNA (sgRNA) with the Benchling CRISPR Design Tool. The sgRNA sequence (5′- GATGCTTCCACCTCTCACCA-3′) was designed to target exon 3 of the *IRF1* gene. The sgRNA was subcloned into the pX459 plasmid (Addgene plasmid #62988) and used to transfect HeLa cells with JetPRIME (Polyplus 114–07) according to the manufacturer's instructions. Clones were selected using puromycin resistance, and single cells were expended and verified by Western blotting analysis and genomic sequencing for the KO. Similarly, *JAK1* KO and double *IFNAR1/IFNAR2* KO cell lines were generated as previously described (11, 12, 100).

### IRF1 and GFP overexpression

Transient transfections of HeLa cells were performed with JetPRIME reagent (Polyplus 114–07) according to the manufacturer's protocol. Forty-eight hours later, the cells were treated to assess antiviral activity and gene expression, and protein phosphorylation was determined by Western blotting analysis. Constructs used are as follows: IRF1: Full-length protein inserts in mammalian vector pDisplay; GFP: Full-length protein inserts in mammalian vector pDisplay.

### Western blot analysis

For the WB analysis, cells were lysed in PBS (pH 7.4) supplemented with 1% NP-40, 1 mM EDTA, and a mix of protease inhibitors (Sigma P8340), phosphatase inhibitor cocktail 2 (Sigma P5726), and phosphatase inhibitor cocktail 3 (Sigma P0044). The lysates were separated using 4 to 20% SDS-PAGE (GenScript M00657) and transferred onto a 0.45 μm nitrocellulose membrane (Bio-Rad). Membranes were blocked with 5% bovine serum albumin before primary antibody incubation. Detection of specific protein bands was carried out using enhanced chemiluminescence substrate. The primary and secondary antibodies used are listed in Table S2, including HRP-conjugated anti-mouse (Jackson ImmunoResearch, 115035146) and HRP-conjugated anti-rabbit (Jackson ImmunoResearch, 111035144). The WBs were visualized using the Odyssey Fc imaging system (LI-COR Biosciences). Band intensities were quantified with Image Studio Lite software (LI-COR Biosciences). For normalization, the following steps were applied: 1) The highest signal intensity band on each membrane was set as the reference with a value of 1. 2) Other bands on the same membrane were normalized by dividing their intensities by this reference value. 3) To normalize across multiple membranes, each band’s intensity detected by a particular antibody in each experiment was further divided by the normalized value of the loading control, tubulin. 4) For phosphorylated proteins, the intensity of each band was additionally normalized by dividing the phosphorylated protein's intensity by the intensity of the corresponding total protein.

### Antiviral and antiproliferative assays

HeLa cells (1.2 × 10^4^ cells for antiviral assays and 2 × 10^3^ cells for antiproliferative assays) were seeded into flat-bottomed 96-well plates and cultured overnight. For both assays, cells were treated with ten 3-fold serial dilutions of either IFN-β or IFN-γ. For IFN-β, the starting concentrations were 500 pM for the antiviral assay and 50 nM for the antiproliferative assay, with cells incubated for 4 h prior to virus addition. For IFN-γ, the starting concentrations were 500 PM for the antiviral assay and 50 nM for the antiproliferative assay, with an 8-h incubation before virus addition in the antiviral assay. Antiviral protection against VSV and EMCV was evaluated by assessing the inhibition of virus-induced cytopathic effects. After the respective incubation periods with interferons, VSV or EMCV were introduced into the wells, followed by an 18-h incubation for VSV or 20 h for EMCV. Antiproliferative activity was measured 96 h post-treatment with either IFN-β or IFN-γ. Cell viability was determined through crystal violet staining in a 96-well plate format, with absorbance readings at 590 nm using a TECAN INFINITE M PLEX plate reader, capturing 5 × 5 multiple reads per well. Data normalization was performed using the formula:

$$Normalized\;Value=\frac{\left(Sample-Negative\;control\right)}{\left(Positive\;control-Negative\;control\;\right)}$$

where "Sample" refers to treated cells exposed to the virus, "Negative Control" refers to cells exposed to the virus without treatment, and "Positive Control" refers to NT cells without virus exposure. EC50 values and cell sensitivity to the treatments were calculated by fitting the response curves using GraphPad Prism software (version 9.5.0.730).

### Intrinsic antiviral protection

HeLa cells (2 × 10^4^) were seeded into flat-bottomed 96-well plates and cultured overnight. To assess the cells’ intrinsic antiviral capacity, either VSV or EMCV was added directly to the wells without any prior treatment. Cells were incubated with VSV for 8 h or EMCV for 12 h. Following incubation, cell viability was determined by crystal violet staining, as described in the antiviral and antiproliferative assay section. Absorbance was measured at 590 nm using a TECAN INFINITE M PLEX plate reader, and data were normalized accordingly.

### Quantitative PCR analysis

The relative expression levels of selected human ISGs were determined using the Applied Biosystems ViiA 7 Real-Time PCR System, following previously established protocols (5, 11, 100). PCR reactions were performed using Fast SYBR Green Master Mix (Applied Biosystems), and cDNA was synthesized from 1 μg of total RNA using the high-capacity cDNA reverse transcription kit (Applied Biosystems). Total RNA was extracted with the NucleoSpin RNA kit (Macherey-Nagel). For qPCR, 14.5 ng of cDNA was used per reaction, with a total reaction volume of 7 μl, using the appropriate primers listed in Table S1. Relative expression levels were calculated using the ΔΔCT (cycle threshold) method, with the fold-change expression determined using the formula RQ = 2^∧^−ΔΔCT. Hypoxanthine-guanine phosphoribosyltransferase 1 served as the reference gene for normalization across samples.

### RNA-seq sample preparation, library preparation, and sequencing

RNA-seq analysis was performed on HeLa wildtype (WT) cells, *IRF1* KO cells, and cells with OE of IRF1 or GFP as a control. All the samples were done in two biological duplicates. The specific conditions included:

WT HeLa cells (NT) for basal RNA expression.

WT HeLa cells treated with 2 nM IFN-β for 16 h.

WT HeLa cells treated with 100 nM IFN-γ for 6 and 16 h.

*IRF1* KO cells (NT).

*IRF1* KO cells treated with 100 nM IFN-γ for 6 and 16 h.

WT HeLa cells overexpressing IRF1.

*JAK1* KO cells overexpressing IRF1.

*IFNAR*s KO cells overexpressing IRF1.

WT HeLa cells overexpressing GFP as a control for transfection.

RNA was isolated as described for qPCR analysis. Library preparation and RNA-seq experiments were conducted at the INCPM units at the Weizmann Institute of Science. Samples were sequenced using the Illumina NovaSeq SP platform with 100-cycle runs. Library preparation included the addition of unique molecular identifiers to each DNA fragment during reverse transcription using an oligo dT primer. Unique molecular identifiers are short molecular tags that help to reduce errors and quantitative bias during PCR amplification by uniquely labeling each original RNA molecule before amplification, following protocols described in previous studies (101, 102).

### RNA-seq data analysis

RNA-seq data were analyzed using the UTAP, a robust pipeline developed by the Weizmann Institute of Science for high-throughput transcriptomic analysis (60), The UTAP pipeline performs a comprehensive workflow, including quality control, read alignment, transcript quantification, and differential expression analysis. Raw sequencing reads were first assessed for quality using FastQC to identify any issues in read quality, adapter content, or sequence duplication levels. Reads were then aligned to the human genome reference (hg38) using STAR (103), a highly efficient RNA-seq aligner. The UTAP pipeline automatically handles the alignment process, mapping reads to annotated gene models and providing high-quality alignments for subsequent analysis. Differentially expressed genes (DEGs) were identified and normalized using DESeq2 (61). Three thresholds were applied to determine significant DEGs: 1) an adjusted *p*-value (p-adj) of ≤ 0.05, 2) a log_2_ fold change of ≥ 2 or ≤ −2, and 3) a base mean value above 5 to ensure that all samples exhibited a minimum level of expression.

### Heatmap generation and clustering analysis

RNA-seq data were analyzed to identify expression patterns and classify genes into distinct clusters. Hierarchical clustering and heatmap visualization were performed using Python libraries, including pandas (104), numpy (105), scipy (106), scikit-learn (107), matplotlib (108), and seaborn (109). RNA-seq data were subjected to hierarchical clustering using Ward’s method, implemented with the linkage function from the scipy library. A dendrogram was generated to visualize the clustering relationships among samples, indicating the similarity of expression profiles. Based on the dendrogram, a flat clustering was performed using the fcluster function, dividing the data into four clusters. Heatmap of median expression with annotations: For each identified cluster, the median expression, IQR, and standard deviation were calculated. These values were visualized in a heatmap with cluster-specific annotations, displaying median expression values alongside their respective IQR and standard deviation. Cluster labels included the number of genes in each cluster, providing additional context.

### Pathway analysis using QIAGEN IPA

To interpret the biological significance of RNA-seq results, we performed pathway analysis using QIAGEN IPA (62). Genes from the RNA-seq clustering analysis were uploaded to the IPA software, where they were mapped to known molecular networks and pathways. The analysis focused on identifying pathways, upstream regulators, and interaction networks associated with DEGs. For inclusion in the IPA analysis, genes were selected based on a threshold of an adjusted *p*-value ≤ 0.05 and a log_2_ fold change ≥ 2 or ≤ −2, as described in the IPA user guide. These criteria ensured that only genes with statistically significant changes in expression and meaningful fold changes were considered in the analysis. The IPA software generated a Graphical Summary, which provides an overview of key pathways, predicted regulators, and molecular interactions derived from the RNA-seq data.

### LEGENDplex 5-plex cytokine assay

In this study, we used the LEGENDplex 5-Plex kit (Cat. 741271) to measure cytokine levels in conditioned media, following the manufacturer's protocol (69). Conditioned media were collected from target cells 48 h post-transfection and incubated with cytokine-specific capture beads for 1 h. After incubation, samples were washed and analyzed using flow cytometry (Cytoflex S, Beckman Coulter). The data obtained from the flow cytometry analysis were processed using the LEGENDplex software, which facilitated quantification of the cytokines. Appropriate controls, as specified by the manufacturer, were included in each assay to ensure accurate and reliable results.

### Preparation and analysis of conditioned media

Conditioned media (CM) from WT and *IFNAR*s KO cells. 17 ml of CM was collected from each cell type. The CM was concentrated using an Amicon Ultra centrifugal filter unit with a 10 kDa molecular weight cutoff, reducing the volume to 500 μl. The concentrated CM was then subjected to size-exclusion chromatography using a Superdex 200 10/300 Gl gel filtration column (GE, cat. 28–990944). Fractions of 1 ml each were collected, and the antiviral activity of each fraction was tested for its ability to protect against VSV infection, using a protocol identical to that employed for IFN-β, except for the use of CM as the treatment.

### Mass spectrometry

Samples were digested with trypsin overnight at 37 °C and analyzed using a Vanquish liquid chromatography system (Thermo Scientific) coupled to a timsTOF SCP mass spectrometer equipped with a CaptiveSpray ion source (Bruker Daltonics). The mass spectrometer was operated in positive data-dependent acquisition mode. One microliter of the peptide mixture was injected by an autosampler onto a C18 trap column (Pepmap Neo C18, 5 μm, 0.3 × 5 mm, Thermo Scientific). After trapping, peptides were eluted from the trap column and separated on a C18 analytical column (Pepsep C18, 150 × 0.15 mm, 1.5 μm, Bruker Daltonics) using a linear gradient of 5% (v/v) to 35% (v/v) acetonitrile in water over 35 min, with a flow rate of 1.5 μl/min. Both the trap and analytical columns were maintained at 50 °C. The timsTOF SCP was configured using standard proteomics PASEF (parallel accumulation-serial fragmentation) parameters. The target intensity per individual PASEF precursor was set to 20,000, with an intensity threshold of 1500. The ion mobility scan range was set between 0.6 and 1.6 V s/cm^2^ with a ramp time of 100 ms, and 10 PASEF MS/MS scans were acquired per cycle. Precursor ions in the m/z range of 100 to 1700, with charge states between 2+ and 6+, were selected for fragmentation. Active exclusion was enabled for 0.4 min. Raw data were processed using PeaksStudio 10.0 software (Bioinformatics Solutions). Search parameters included trypsin (semispecific) as the enzyme, with carbamidomethylation set as a fixed modification and oxidation of methionine and acetylation of protein N terminus as variable modifications. The data were searched against a local database containing interferon sequences and against Human Proteome for MS database. False discovery rates (FDRs) were estimated using the target–decoy approach implemented in PeaksStudio. Peptide-spectrum matches were filtered to achieve an FDR of ≤1%. Peptides were accepted if they passed a peptide-level FDR threshold of ≤1% and were supported by high-confidence peptide-spectrum matches only.

### IRF1 DBD-mNeonGreen

Expression of IRF1 DBD-mNeonGreen was done in pET28-*IRF1* DBD-mNeonGreen within T7 competent bacterial cells. The transformed bacteria were cultured in 2 YT media supplemented with kanamycin for selection. After growth, the bacterial cells were harvested and lysed using sonication in PBS buffer containing 360 mM NaCl, 10% glycerol, protease inhibitors, and DTT to prevent protein degradation. Following lysis, the lysate was clarified by centrifugation and further filtered using a 0.45 μm filter. The filtered lysate was then loaded onto a Hi-Trap SP HP anion exchange column (GE Healthcare, Cat. 17115101) for initial purification. The final purification step involved size-exclusion chromatography using a Superdex 75 Increase 16/600 column (GE Healthcare, Cat. 28–989333) to ensure complete purification and separation of the IRF1 DBD-mNeonGreen protein.

After protein purification, three different samples were prepared to assess DNA-binding specificity. The first sample contained purified protein without any DNA. The second sample included the purified protein incubated with a synthesized DNA oligonucleotide containing a specific IRF1 binding sequence (5′-GAGAAGTGAAAGTACTTTCACTTCTC-3′) at a 1:1 M ratio of protein to DNA. The third sample consisted of the purified protein with a scrambled control DNA sequence (5′-ATATTACGTCGCACTAGGATAGATCT-3′), also added at a 1:1 M ratio. All samples were incubated for 4 h at room temperature before measurement to ensure sufficient binding interaction. These samples were then used to evaluate the protein's specific binding affinity and interaction with DNA in subsequent assays.

### Size-exclusion chromatography

A sample was manually injected onto a Superdex 200 Increase 10/300 Gl column (GE, cat. 28–990944). The column was pre-equilibrated with PBS at pH 7.4, supplemented with 360 mM NaCl and DTT. To determine the molecular mass of the eluted proteins, a standard curve from a previous study (110) was used.

### Mass photometry

Microscope coverslips (no. 1.5, 24 × 50, cat# 0107222, Marienfeld) were cleaned by sequential sonication in 50% isopropanol (HPLC grade)/Milli-Q water and Milli-Q water alone for 5 min each, followed by drying with a nitrogen stream. Four gaskets (Reusable culturewell gaskets, 3 mm diameter × 1 mm depth, cat# GBL103250–10 EA, Sigma-Aldrich) were cut into a 2 × 2 array, cleaned using the same method as the coverslips, and placed on top of the coverslip, with each well used for a separate sample measurement. Immediately before mass photometry measurements, protein stocks were diluted in PBS at pH 7.4. A fresh buffer was added to each well to find the focal position, which was then secured using an autofocus system based on total internal reflection for consistent focus throughout the measurements. For each measurement, 5 μl of diluted protein solution (at nanomolar concentrations) was added to the well, and after the autofocus system stabilized, 120-s movies were recorded. Each sample was measured in triplicate. Calibration of the contrast-to-mass conversion was performed in the same measurement buffer using urease (Sigma cat# U7752-1VL) as a reference, with known oligomer masses. All data were acquired using a OneMP mass photometer (Refeyn Ltd, Oxford). Data collection was done using AcquireMP software (v2.2, Refeyn Ltd), and data analysis was performed with DiscoverMP software (v2.3.0, Refeyn Ltd).

### Nano DSF melting assay

Protein thermal stability was assessed using the Tycho NT.6 instrument (NanoTemper) (111). Protein samples were prepared at a concentration of 0.1 to 0.5 mg/ml in PBS. A total of 10 μl of each protein sample was loaded into Tycho NT.6 capillaries and inserted into the instrument. Thermal unfolding was monitored by heating the samples from 35 °C to 95 °C while recording fluorescence at 330 nm and 350 nm. The fluorescence ratio (350/330 nm) was used to generate unfolding curves, and the melting temperature (Tm) was determined as the inflection point of the curve, indicating the temperature of 50% protein unfolding.

### Protein binding microarrays

Experiments were conducted as previously described (72, 73). Briefly, custom-designed DNA microarrays (Agilent Technologies) were used, comprising ∼15,000 unique single-stranded 60-base oligonucleotides. The DNA library used here consists of de-Bruijn sequences representing all possible 9-mers and a constant 3′ end. The arrays were double stranded through incubation with a primer (complementary to the 3′ end constant region), Thermo Sequenase DNA polymerase, dNTPs, and 10X reaction buffer (Cytiva) for 2 h at a temperature gradient (85 °C to 60 °C). The double-stranded microarray was prewet in PBS 0.01% Triton X-100 for 5 min and blocked with nonfat milk 2% (w/v) for 1 h. Then, it was washed with PBS 0.1% Tween-20 for 5 min with PBS 0.01% Triton X-100 for 2 min. Purified IRF1-mNeonGreen was diluted to a final concentration of 2 μM in a binding mixture (nonfat milk 2%, 51.3 ng/μl salmon testes DNA, 0.2 mg/ml bovine serum albumin, 0.2% Triton X-100, PBS, 360 mM NaCl, and 1 mM DTT). The microarray was incubated for 1 h with the binding mixture at room temperature and then washed with PBS 0.5% Tween-20 for 3 min and with PBS 0.01% Triton X-100 for 2 min. IRF1-mNeonGreen fluorescence was scanned at 488 nm using a GenePix 4400A microarray scanner and the GenePix pro analysis software. For data analysis, signal was first spatially detrended, and position weighted matrices were generated using the "seed and wobble" algorithm (72). The energy-normalized logo for IRF1-mNeonGreen was generated using enoLOGOS (74).

### ChIP-seq data analysis

ChIP-seq raw data (GEO accession number: GSE6928615 and GSE6928616) were reprocessed with a standardized pipeline using the Galaxy (75) platform following the methodologies outlined in (43). Data processing included quality control, read alignment, peak calling, and visualization. Raw reads were aligned to the human genome (hg38) using Bowtie2, followed by peak calling with MACS2 to identify regions of enriched IRF1 binding. Visualization of ChIP-seq coverage and comparison with predicted binding sites was performed using the IGB (76). Identified peaks were cross-referenced with predicted binding affinity scores to confirm potential IRF1 binding sites across the promoter regions of genes identified from RNA-seq analysis.

### Generation of predicted binding scores and BedGraph files

To predict IRF1 binding affinity across promoter regions, we used a custom Python script to calculate z-scores for each 7-mer sequence, based on data from a UniProbe (112) binding affinity file generated from our PBM experiments. The DNA sequence of interest was defined, and the UniProbeZScoreFile parser was used to calculate binding affinity scores for each 7-mer, resulting in a vector of predicted binding scores. Genomic coordinates were assigned according to the target region. A BedGraph file was generated using a custom function, which iteratively mapped each score to its corresponding genomic position with a defined step size, creating a continuous profile of predicted binding affinity across the region. The resulting BedGraph file was used for visualization and comparison with ChIP-seq data in downstream analyses.

### Visualization in the IGB

The generated BedGraph files containing predicted IRF1 binding scores were visualized using IGB (76). ChIP-seq data (GEO accession numbers: GSM6928615, GSM6928616) were loaded alongside the BedGraph tracks to allow direct comparison of predicted binding sites with observed ChIP-seq coverage. The genomic coordinates of each track were adjusted to ensure alignment, facilitating the identification of regions where predicted high-affinity binding sites overlapped with strong ChIP-seq peaks. To aid interpretability, predicted binding scores were transformed using an inverse log_2_ function (2^∧^z-score) within IGB, restoring the relative binding signal to linear scale for more intuitive visualization. This visualization approach enabled a detailed comparison of computational predictions with experimental data, highlighting areas of potential IRF1 binding in the promoter regions of target genes.

### Transcriptomic correlation analysis using the Human Protein Atlas

Transcriptomic data across 1206 human cell types were retrieved from the Human Protein Atlas (https://www.proteinatlas.org/about/download), which provides normalized gene expression values as normalized transcripts per million. Transcripts per million is a unit that reflects relative transcript abundance across samples while accounting for both sequencing depth and gene length. Pairwise correlations were plotted as scatter plots, with HeLa highlighted relative to the distribution of all other cell types.

## Data availability

The RNA sequencing data generated in this study have been deposited in the NCBI GEO database and are accessible under the SuperSeries accession number PRJNA1328237.

## Supporting information

This article contains supporting information.

## Conflict of interest

The authors declare that they have no conflicts of interest with the contents of this article.
