# Supplementary material for: IRF1 is a context-dependent homeostatic gatekeeper of basal immunity and antiviral readiness
Source: J Biol Chem. 2025 Dec 30;302(2):111118. doi: 10.1016/j.jbc.2025.111118 (PMC12887176; doi:10.1016/j.jbc.2025.111118)
Supplement: Supporting information [file mmc1.pdf]

Supplemental Materials

IRF1 is a Context-Dependent Homeostatic Gatekeeper of Basal Immunity and Antiviral Readiness

Authors: Eyal Zoler, Irina Miodownik, Shifra Ben-Dor, Daniel Harari, Jiri Zahradnik, Ariel Afek, Gideon Schreiber

Table S1. Primers used for real-time PCR

Gene	Forward Primer	Reverse Primer
OAS2	CTGATCGACGAGATGGTGAA	TGTTTTCCGTCCATAGGAGC
MX2	GCATCCACCTGAATGCCTAC	ATCATGGCTTTCTGCAAGGA
CXCL10	TCCACGTGTTGAGATCATTGC	TCTTGATGGCCTTCGATTCTG
CXCL11	GCCTTGGCTGTGATATTGTGTG	GGCCTATGCAAAGACAGCGT
IFIT 1	GCCACAAAAAATCACAAGCCA	CCATTGTCTGGATTTAAGCGG
LAMP 3	TGGGAGCCTATTTGACCGTCTC	GCTGACAACTGGAGGCTCTGTT

Table S2. Primary antibodies for western blots

**Protein**	**Epitope**	**Concentration**	**Source**	**Catalogue No.**
STAT1	Total	1:1000	Cell Signalling Technology (CST)	14995S
pSTAT1	Tyr701	1:1000	CST	9167S
STAT2	Total	1:1000	Santa Cruz Biotechnology	sc-1668
pSTAT2	pTyr690	1:1000	CST	4441S
IRF1	Total	1:1000	CST	8478S
Tubulin	Total	1:5000	Sigma-Aldrich	T9026

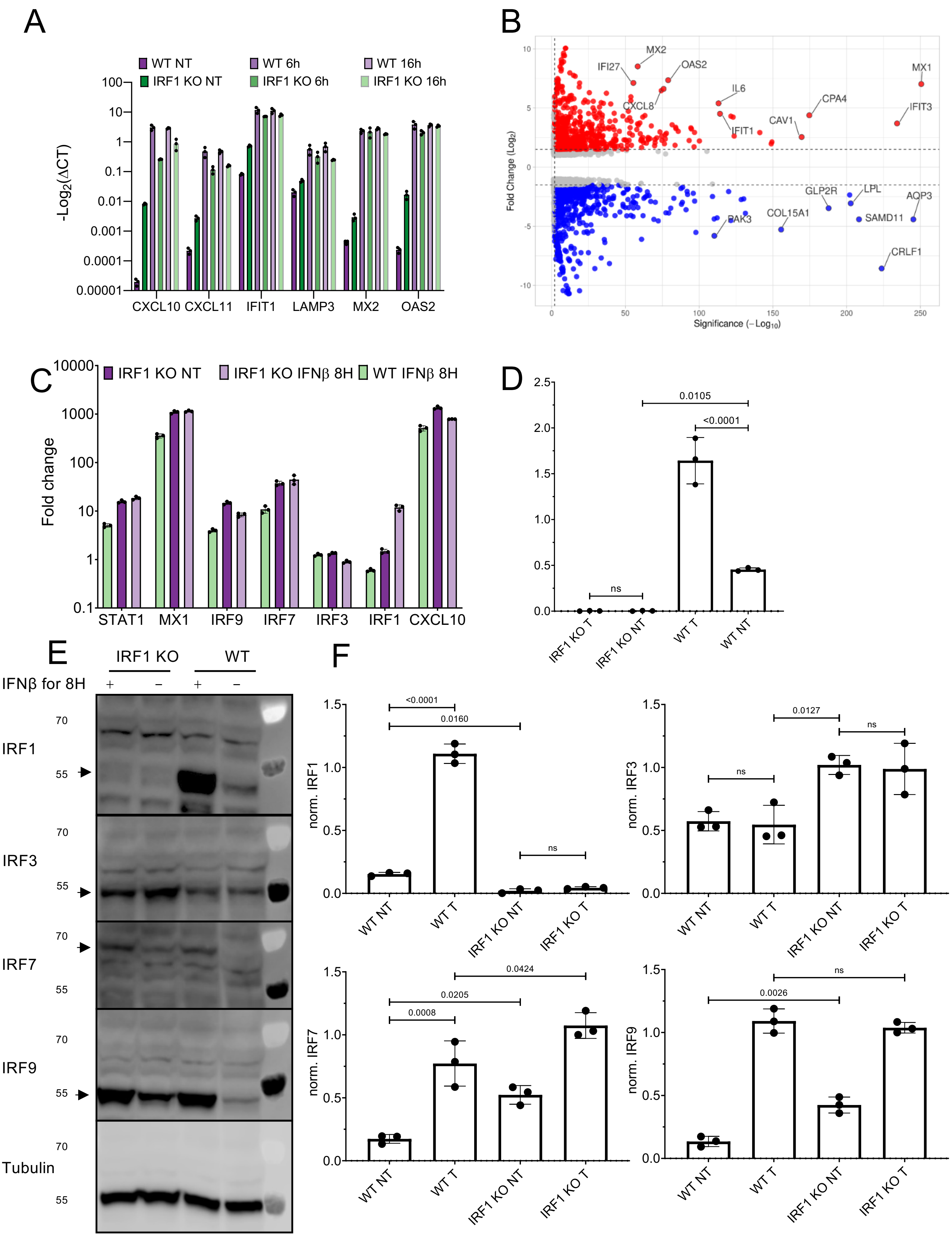
A
WT NT
WT 6h
WT 16h
IRF1 KO NT
IRF1 KO 6h
IRF1 KO 16h
-Log2(ΔCT)
CXCL10 CXCL11 IFIT1 LAMP3 MX2 OAS2
B
Fold Change (Log2)
Significance (−Log10)
MX2
IFI27
OAS2
MX1
IL6
CPA4
CXCL8
IFIT3
CAV1
IFIT1
GLP2R
LPL
AQP3
COL15A1
RAK3
SAMD11
CRLF1
C
IRF1 KO NT
IRF1 KO IFNβ 8H
WT IFNβ 8H
Fold change
STAT1 MX1 IRF9 IRF7 IRF3 IRF1 CXCL10
D
0.0105
<0.0001
ns
IRF1 KO T
IRF1 KO NT
WT T
WT NT
E
IRF1 KO
WT
IFNβ for 8H
IRF1
IRF3
IRF7
IRF9
Tubulin
F
norm. IRF1
norm. IRF3
norm. IRF7
norm. IRF9
<0.0001
0.0160
0.0127
0.0008
0.0205
0.0424
0.0026
ns
WT NT
WT T
IRF1 KO NT
IRF1 KO T

Figure S1. Basal gene expression abundance in *IRF1* KO cells. (A) RNA was extracted from WT and *IRF1* KO cells treated with IFN-β for 6 and 16 hours. The relative abundance of the indicated genes were determined by qPCR and are presented as -log2(ΔCT) normalized to that of *HPRT1*. *XAF1, MX1, MX2*, and *OAS2* are type I IFN induced robust genes; *IDO1, CXCL10* and *CXCL11* are type I IFN induced tunable genes. Data are means + SD of three independent experiments per group. **(B)** Volcano plot from RNA-seq result of *IRF1* KO compared to HeLa WT cells. The Y-axis is log_2 fold change of *IRF1* KO non-treated over WT non-treated and the X-axis is $-log_{10}$ of the Padjusted values. The threshold is log2FC>2 or <-2. (**C**) WT and *IRF1* KO HeLa cells were left untreated or treated with IFN-β for 8 hours, and RNA was extracted for gene expression analysis. The relative mRNA levels of *STAT1, MX1, IRF1, IRF3, IRF7, IRF9*, and *CXCL10* were determined by qPCR. Data are presented as fold change compared with the expression in untreated WT cells and normalized to *HPRT1*. Data are means ± SD of three independent experiments per group. (**D**) Normalized total IRF1 abundance from the WB shown in Fig. 1A (and two more replicates)**.** (**E**) WT HeLa cells and *IRF1* KO cells were left untreated or treated with IFN-β for 8 hours, and whole-cell lysates were analyzed by WB to detect IRF1, IRF3, IRF7, and IRF9 protein abundance. Tubulin was used as a loading control. Data are representative of three independent experiments. (**F**) Quantification of the Western blot signals shown in **(E)**. Band intensities were normalized to tubulin and expressed relative to untreated WT cells. Statistical significance was assessed by one-way ANOVA followed by Tukey's post-hoc test.

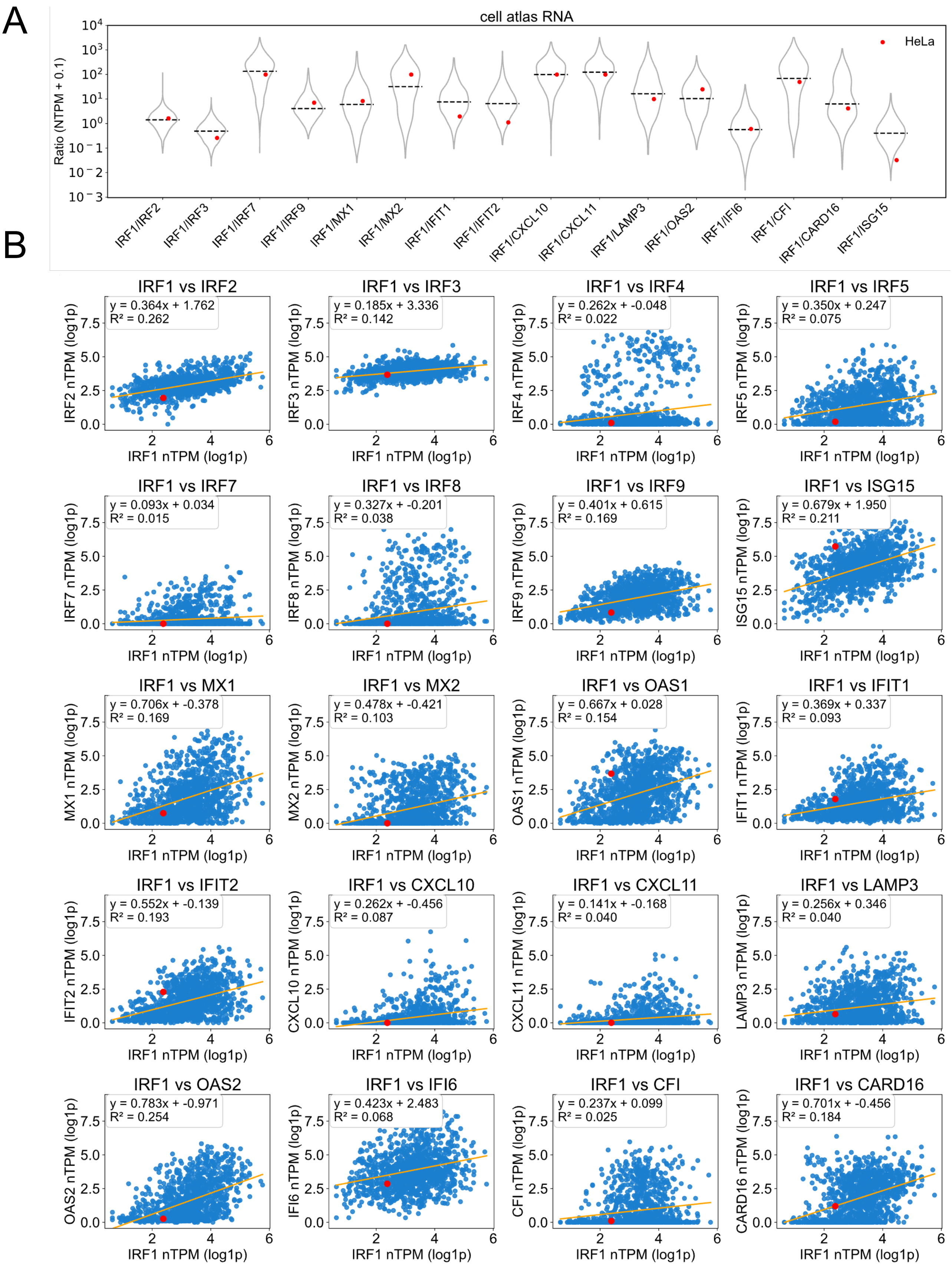

Figure S2. Correlation analysis of IRF1 expression with IRF family members and ISGs across human cell types. (A) Violin plots showing the distribution of gene expression ratios across 1,206 different cell types based on RNA expression data from the Human Protein Atlas (https://www.proteinatlas.org/about/download). Each violin represents log_{10}-transformed normalized Transcripts Per Million (nTPM) ratios per gene, with dashed lines indicating median values. Individual HeLa data points are highlighted in red. **(B)** The x-axis shows log1p abundance of IRF1 and the y-axis is for the analyzed genes. Blue dots represent individual cell types, with HeLa cells being highlighted in red. Orange lines represent linear regression fits, with the corresponding equation and coefficient of determination (R^2).

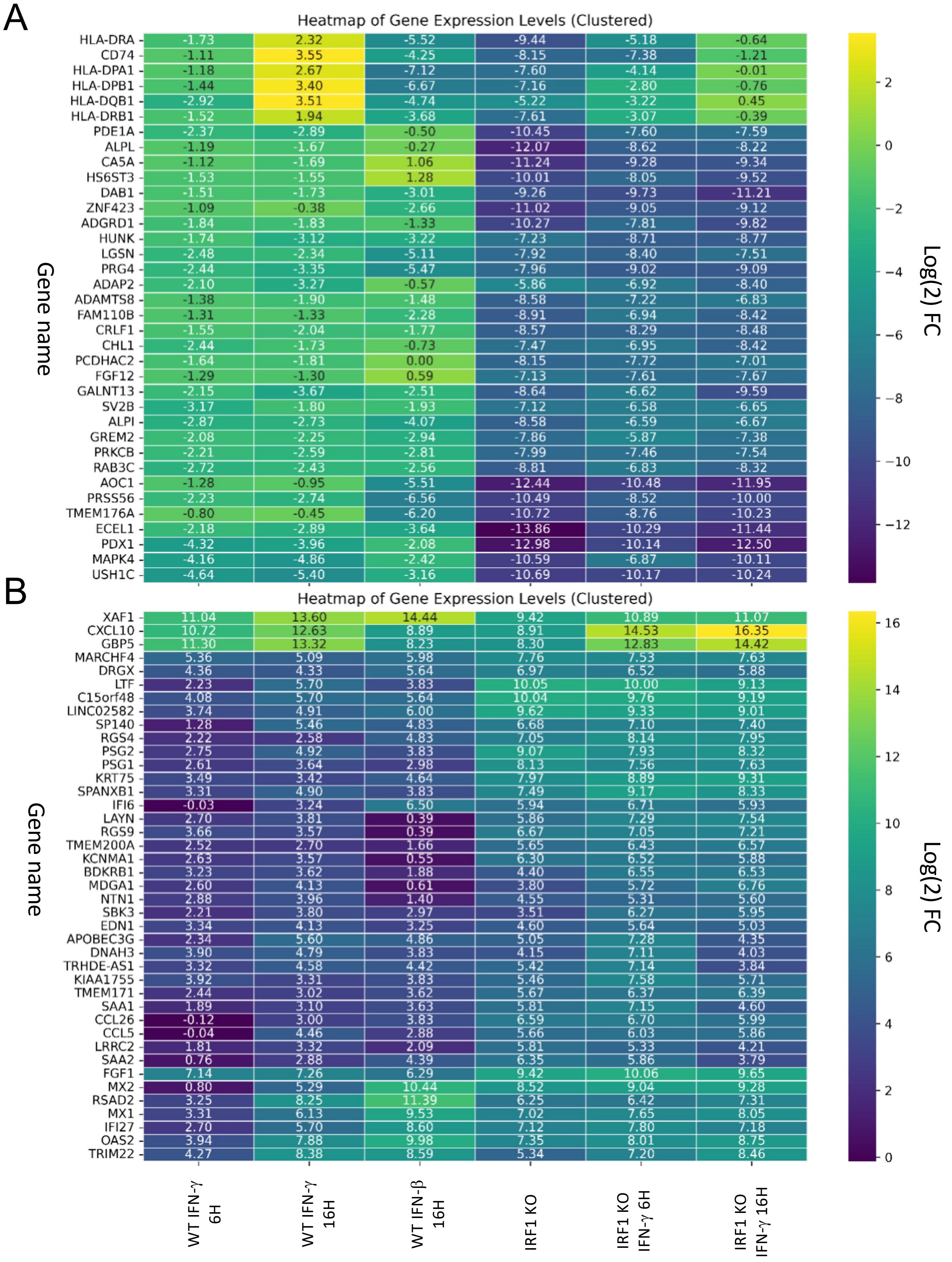

Figure S3. Basal gene expression changes in the *IRF1* KO cells. (A-B) Heatmaps displaying hierarchical clustering of genes from cluster 1 (A) and cluster 4 (B) from Fig. 2C, under the following conditions: WT cells treated with IFN-γ (100 nM) for 6 and 16 hours, WT cells treated with IFN-β (2 nM) for 16 hours, *IRF1* KO cells, *IRF1* KO cells treated with IFN-γ (100 nM) for 6 and 16 hrs. The numbers represent log(2) fold-change relative to WT NT.

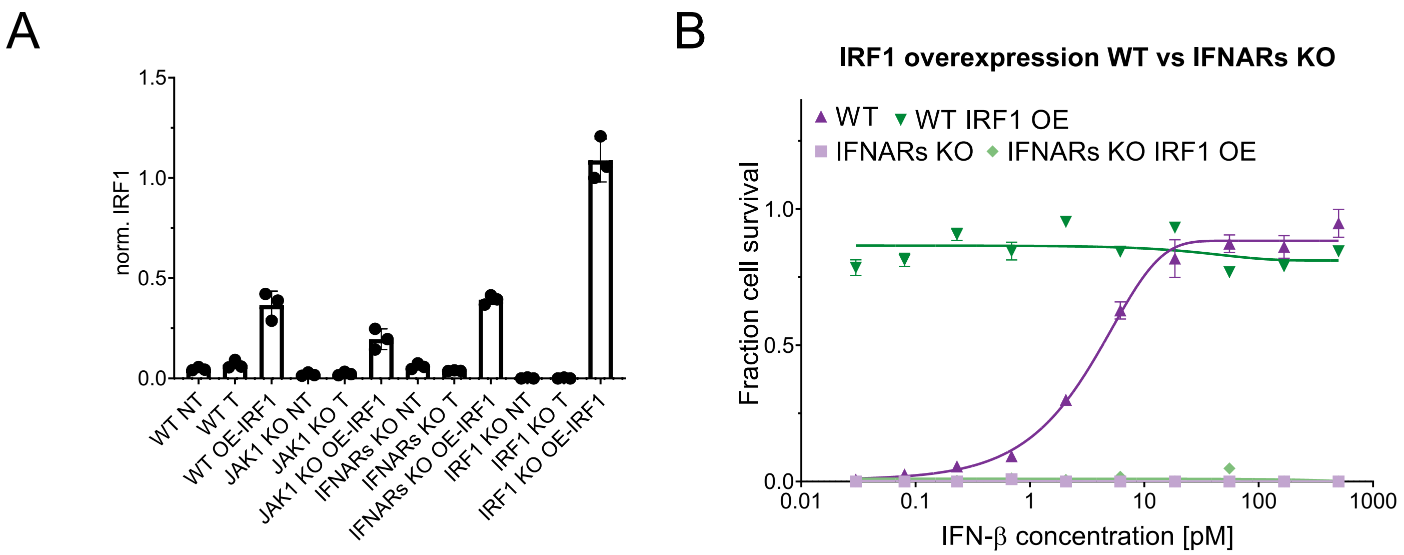

Figure S4. Impact of IRF1 overexpression on STAT signaling, antiviral response, and comparative gene expression ratios across cell lines. (A) Normalized total IRF1 abundance from WB shown in Fig. 3A and two more replicates**. (B)** Antiviral activity against VSV for WT HeLa, *IFNAR* KO and IRF1 OE cells 48 hrs post transient transfection of IRF1. Cells were treated with IFN-β for 4 hrs before infection with the VSV for 18 hrs. Cells were stained with crystal violet for cell viability. Data points are median of 3 independent experiments. Error bars represent the SD.

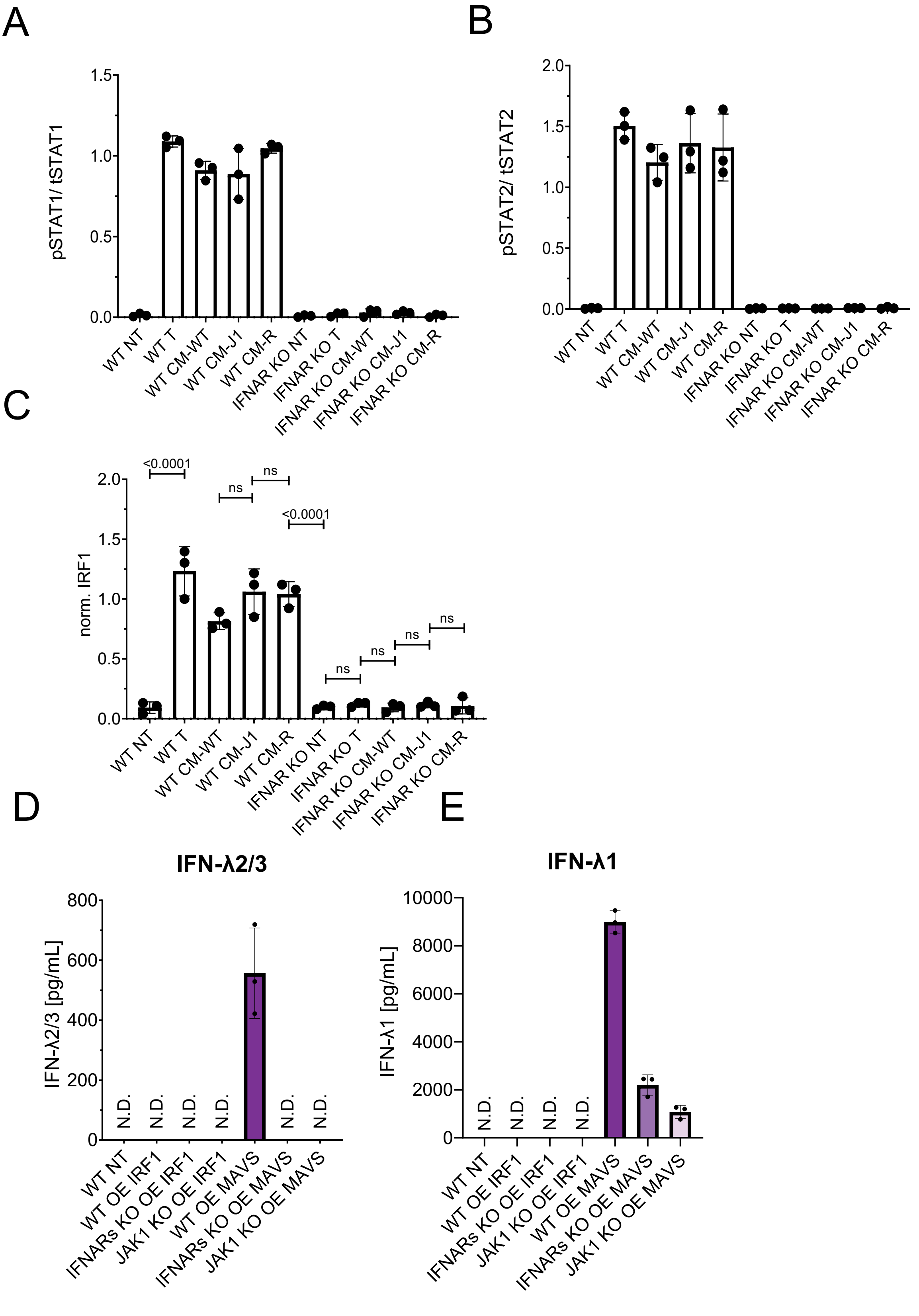

Figure S5. Quantification of cytokine signaling and flow cytometry analysis of IFN abundance in IRF1 and MAVS OE cells. (A-C) Quantification of WB shown in Fig. 4. **(A)** normalized pSTAT1 abundance relative to total STAT. **(B)** Normalized pSTAT2 abundance relative to total STAT2. **(C)** Normalized IRF1 abundance**. (D-E)** Flow cytometry analysis of IFN-λ2/3 **(D)** and IFN- λ1 **(E)** abundance in cells overexpressing IRF1 (IRF1 OE) or MAVS (MAVS OE) in WT, *JAK1* KO, or *IFNAR* KO cells. WT untreated (WT NT) cells were used as a negative control. The data represent the mean +SD from three independent experiments. Statistical significance was assessed by one-way ANOVA followed by Tukey's post-hoc test.

A

> sp P01562 IFNA1_HUMAN Interferon alpha-1

```
  1  MASPFALLMV LVVLSCKSSC SLGCDLPETH SLDNRRTLML LAQMSRISPS SCLMDRHDFG FPQEEFDGNQ FQKAPAISVL
 81  HELIQQIFNL FTTKDSSAAW DEDLLDKFCT ELYQQLNDLE ACVMQEERVG ETPLMNADSI LAVKKYFRRI TLYLTEKKYS
161  PCAWEVVRAE IMRSLSLSTN LQERLRRKE
```

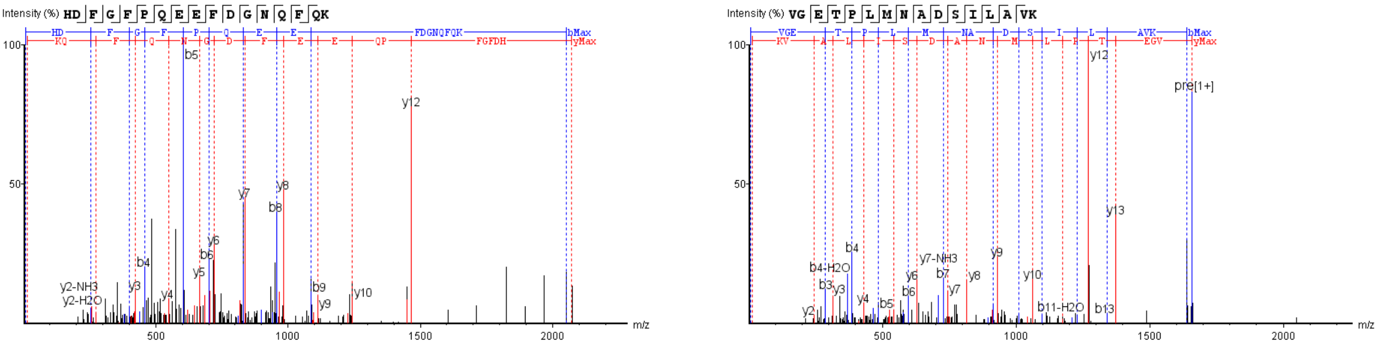

B

> sp P05014 IFNA4_HUMAN Interferon alpha-4 OS=Homo sapiens OX=9606

```
  1  MALSFSLLMA VLVLSYKSIC SLGCDLPQTH SLGNRRALIL LAQMGRISHF SCLKDRHDFG FPEEEFDGHQ FQKAQAISVL
 81  HEMIQQTFNL FSTEDSSAAW EQSLLEKFST ELYQQLNDLE ACVIQEVGVE ETPLMNEDSI LAVRKYFQRI TLYLTEKKYS
161  PCAWEVVRAE IMRSLSFSTN LQKRLRRKD
```

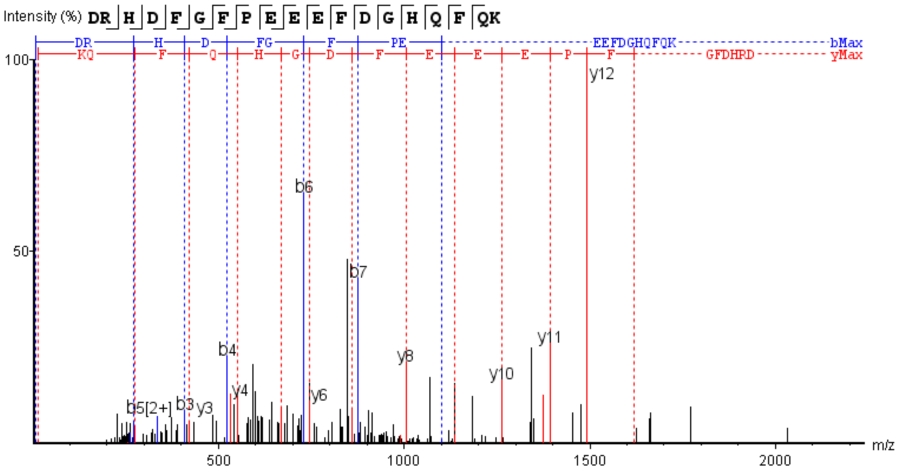

Figure S6. Mass spectrometry profile of protein fractions purified from conditioned media of WT cells. (A) Two unique peptides were identified as belonging to IFNα1. **(B)** One unique peptide was identified as belonging to IFNα4.

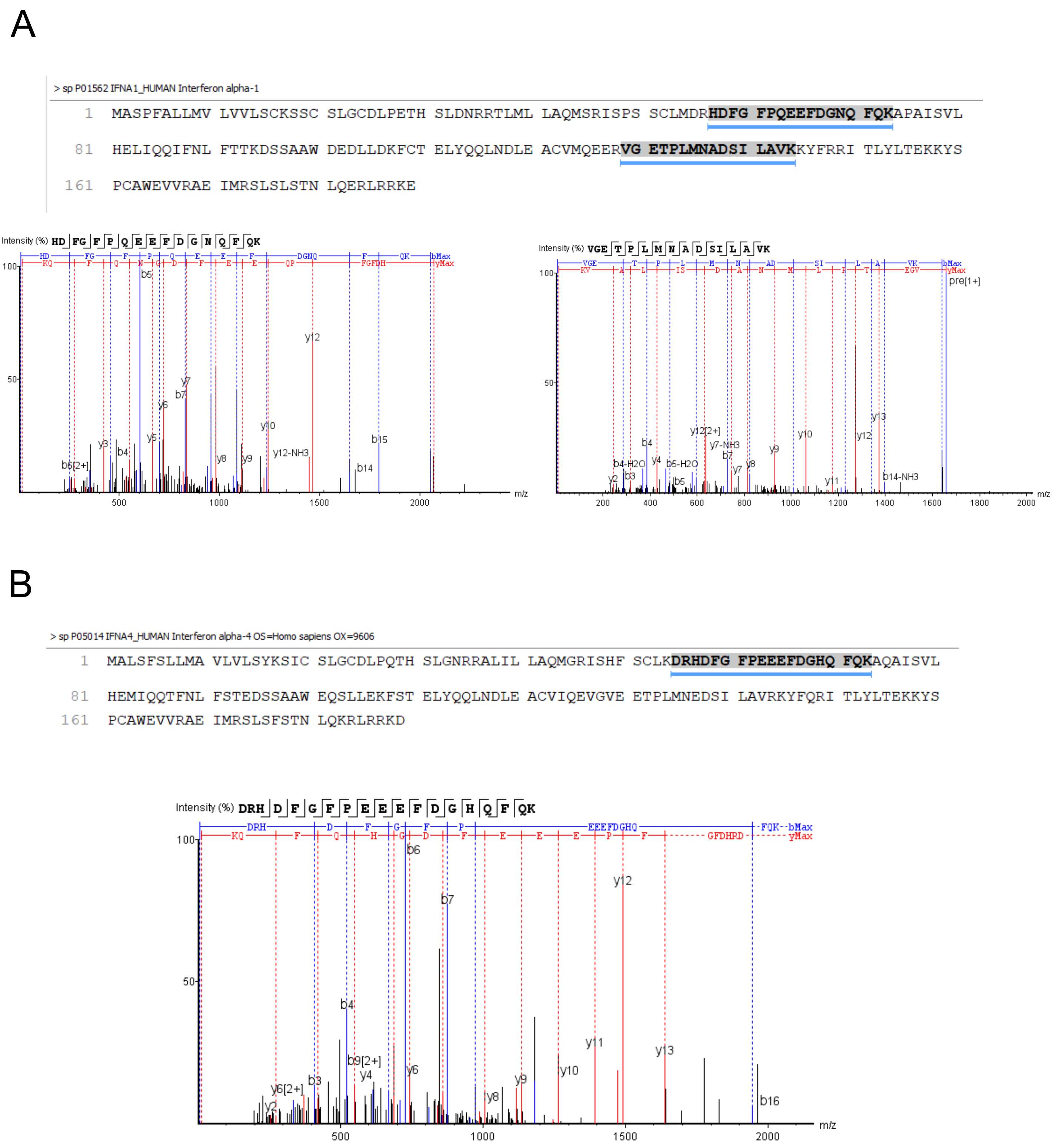

Figure S7. Mass spectrometry profile of protein fraction purified from conditioned media of *IFNAR*s KO Cells. (A) Two unique peptides were identified as belonging to IFNα1. **(B)** One unique peptide was identified as belonging to IFNα4.

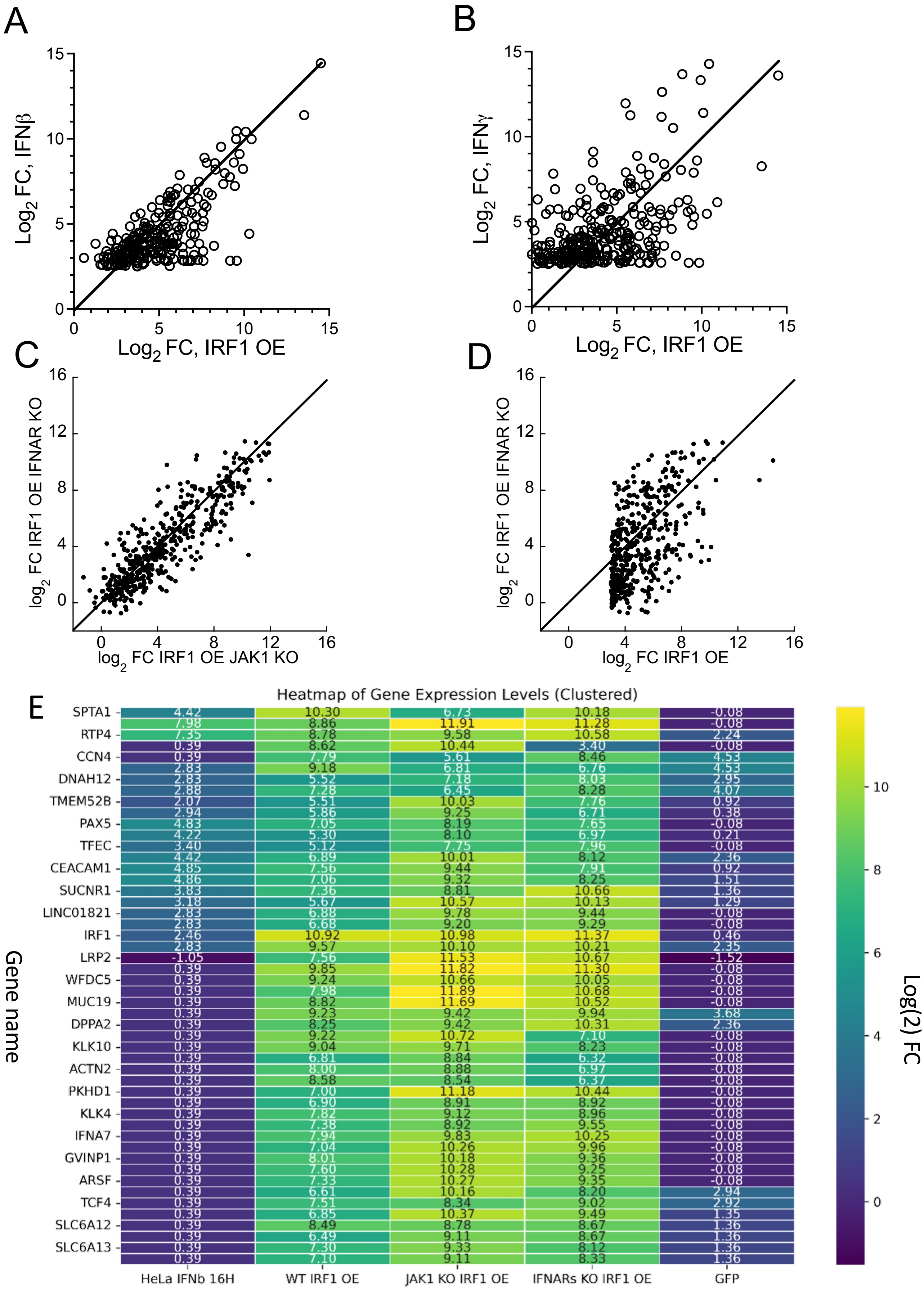

Figure S8. Gene transcript abundance in IRF1 OE cells. (A, B) Genes with FC (relative to WT) of >Log$_2$ 2.5 upon treatment with 2 nM IFN-β for 16 h **(A)** or 100 nM IFN-γ for 16 h **(B)** plotted against FC of IRF1 OE cells. **(C)** FC of gene transcript abundance of IRF1 OE cells relative to WT HeLa cells in background of *JAK1* KO versus *IFNAR* KO. Shown are genes with Log$_2$ FC of > 3 in IRF1 OE cells. **(D)** shows the same gene set as in **(C)** but of abundance upon IRF1 OE versus IRF1 OE in the background of IFNAR KO cells. **(E)** Heatmap of genes extracted from IRF1 OE in WT HeLa cells taken from cluster 3, Fig. 5A under the following conditions: WT IRF1 OE, *JAK1* KO IRF1 OE, and *IFNAR* KO IRF1 OE, as well as WT cells treated with IFN-β (2 nM) for 16 hrs and WT GFP OE used as a transfection control. The numbers represent log(2) fold-change relative to WT NT.

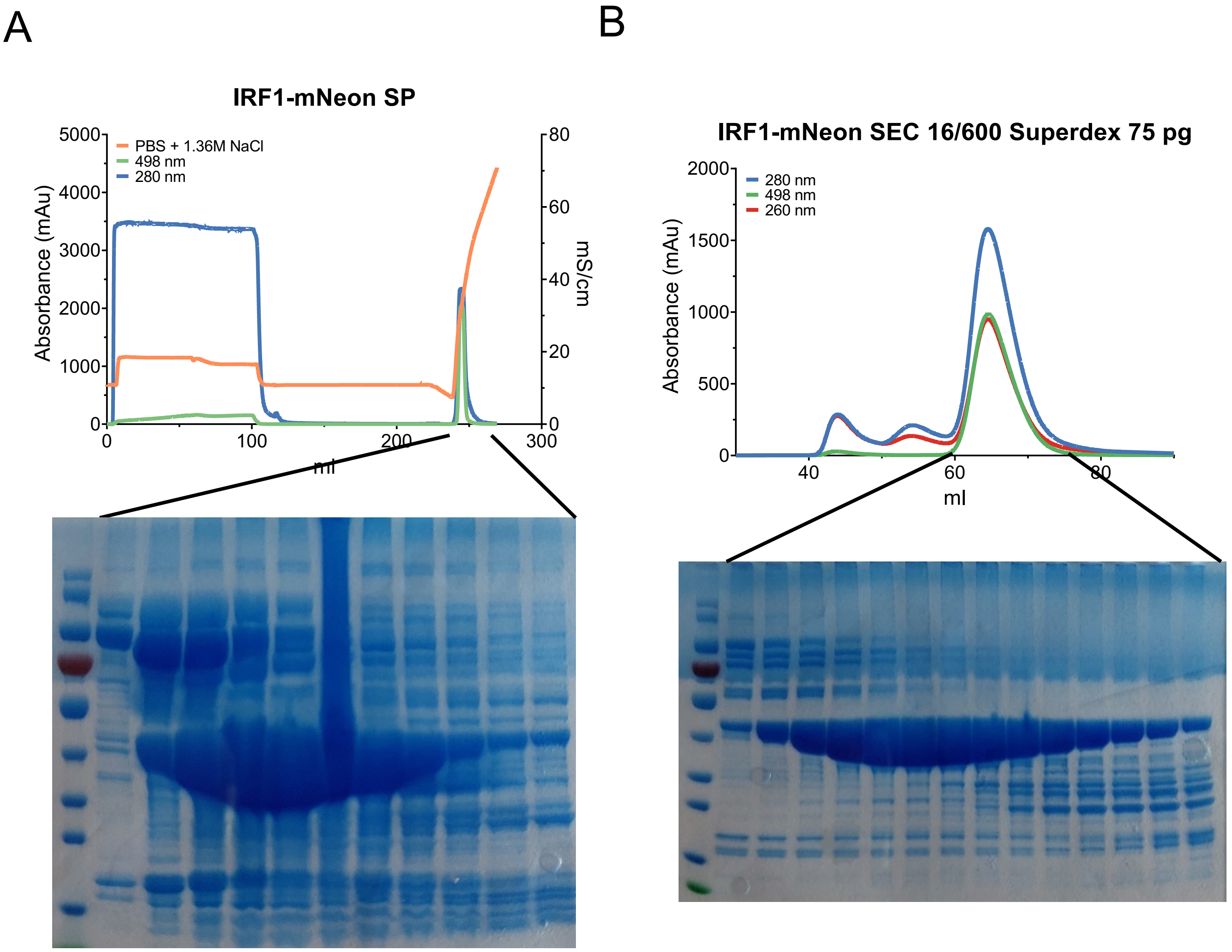

Figure S9. IRF1-mNeon fusion protein purification. (A) IRF1-mNeon was purified using HiTrap SP HP cation exchange chromatography. The blue line show absorbance at 280 nm, the green line 498 nm and the orange line show salinity in mS/cm. **(B)** Size exclusion dendrogram of IRF1-mNeon in SEC (superdex 75pg 16/600 chromatography column). The blue line shows absorbance at 280 nm, the red line 260 nm, and the green line 498 nm.

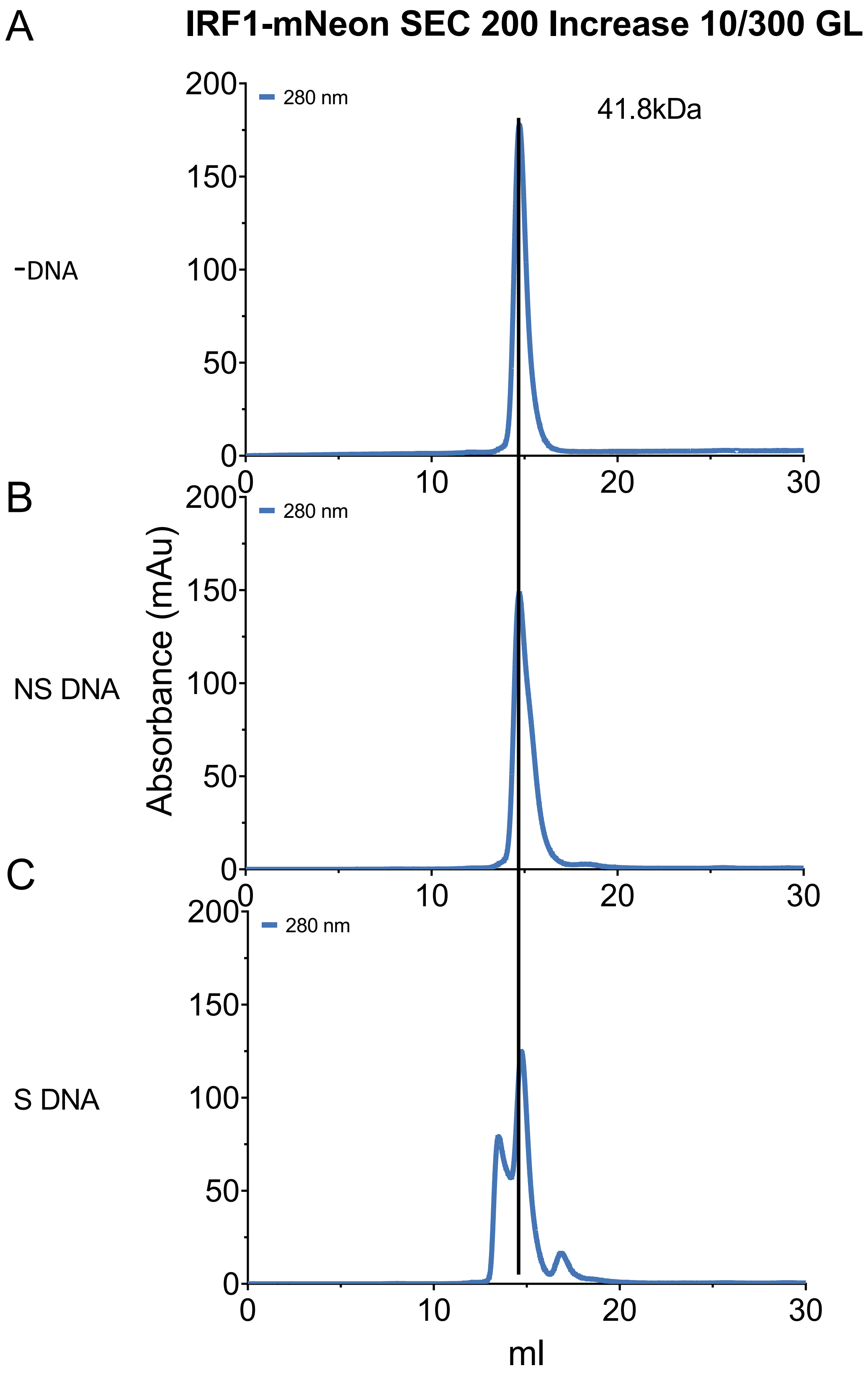

Figure S10. Size exclusion chromatography analysis of IRF1-mNeon complexes. (A) –DNA, SEC of IRF1 protein without DNA. **(B)** NS DNA, IRF1 protein incubated for 4 hours with scrambled DNA. **(C)** S DNA, IRF1 protein incubated for 4 hours with a specific DNA. The blue line shows absorbance at 280 nm and the cross-black line show the elution volume of a protein with MW of 41.8 kDa

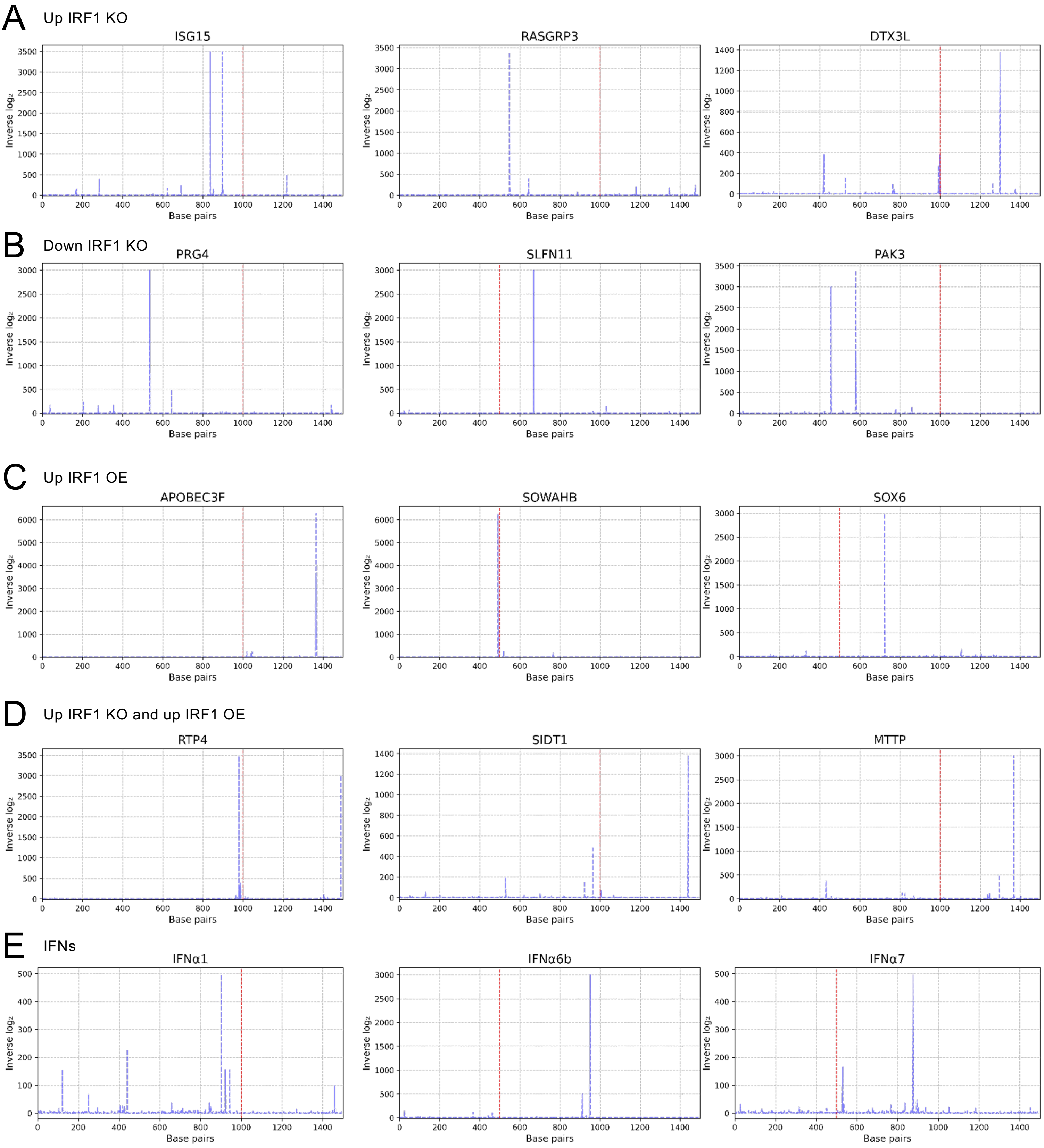

Figure S11. Promoter binding of IRF1 as predicted by our model. (**A-E**) Predicted IRF1 binding peaks across the promoter regions of selected genes that showed significant changes in abundance in the RNA-seq analysis. The promoter regions analyzed are of 1,000 base pairs upstream and 500 base pairs downstream of the transcription start site, marked in red for each gene. Calculated binding affinity is shown as inverse $\log_2$-transformed z-scores (2^z-score), providing a linear-scale representation of predicted IRF1 DNA interaction strength. (**A**) Increased abundance in the *IRF1* KO. (**B**) Decreased abundance in the *IRF1* KO. (**C**) Increased abundance in IRF1 OE. (**D**) Genes with increased abundance in both *IRF1* KO and IRF1 OE. (**E**) Calculated IRF1 binding sites in promoter regions of type I IFN genes.

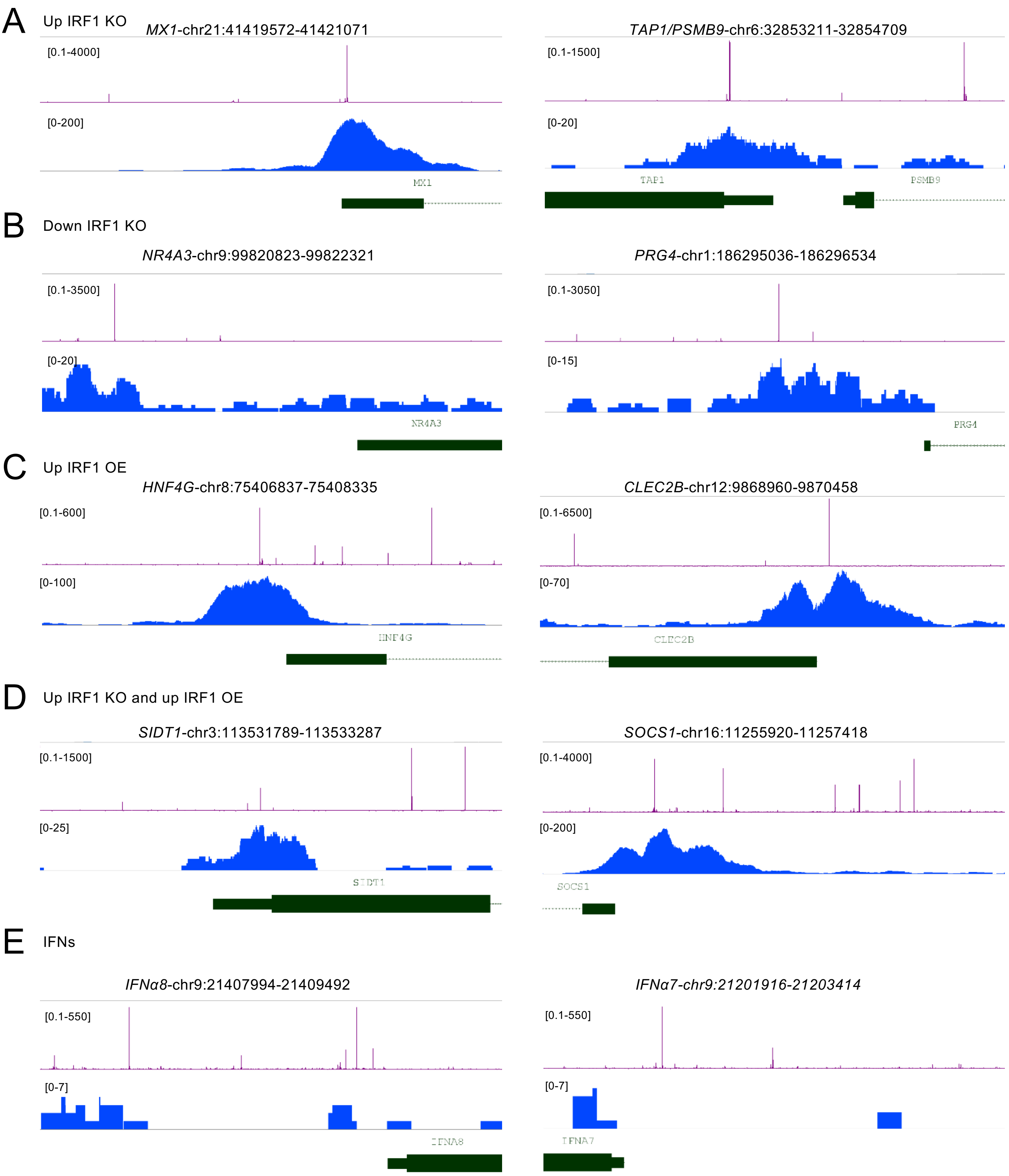

Figure S12. Predicted and observed IRF1 binding in promoter regions of key immune-related genes. (**A-E**) Comparison of predicted IRF1 binding affinity and ChIP-seq signal in promoter regions of genes identified to be controlled by IRF1 according to RNA-seq analysis. The promoter regions span from 1,000 basepairs upstream to 500 basepairs downstream of the transcription start site. Predicted IRF1 binding affinity is shown in purple and was visualized using inverse $\log_2$-transformed z-scores (2^z-score) to represent the relative strength of predicted interactions on a linear scale. ChIP-seq data, reanalyzed from GEO datasets (GSM6928615, GSM6928616), are represented in blue, showing IRF1 binding coverage across the promoter regions. Gene annotations are highlighted in green.

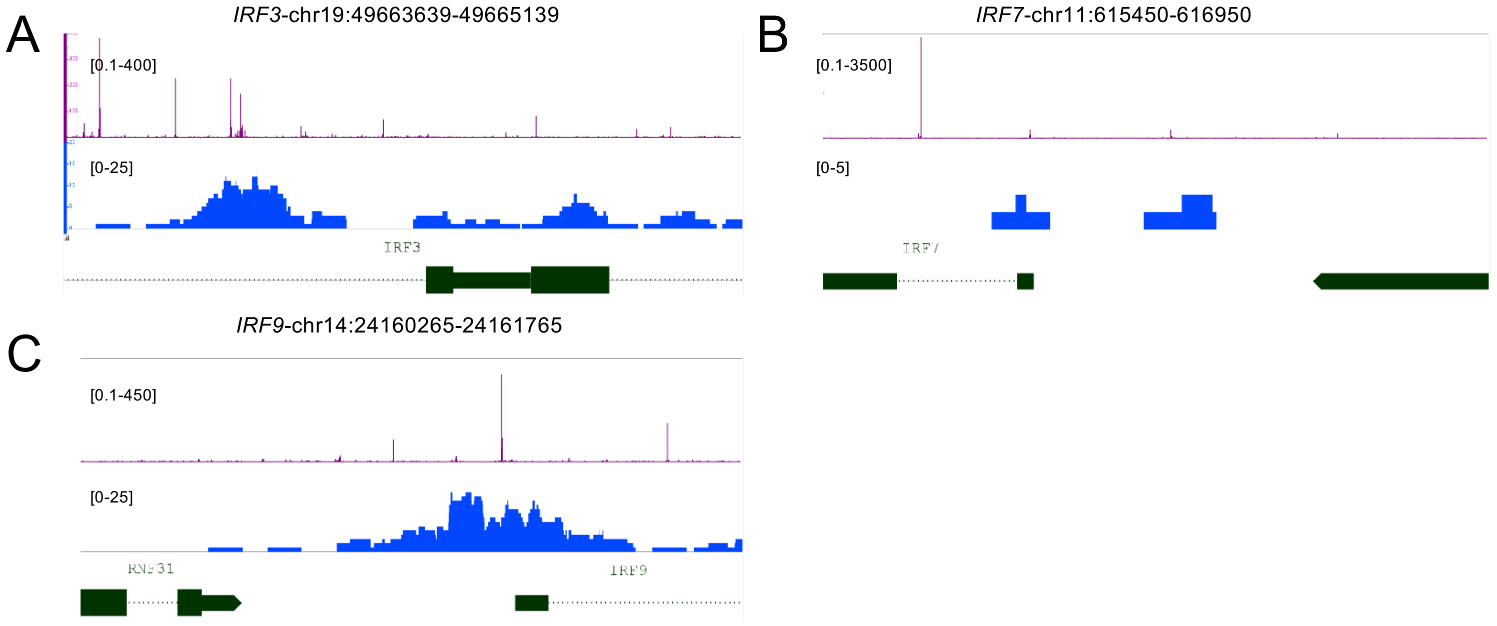

Figure S13. IRF1 binding in promotors of IRF3, IRF7 and IRF9. (A-C) Comparison of predicted IRF1 binding affinity and reanalyzed ChIP-seq coverage in the promoter regions of IRF3 (**A**), IRF7 (**B**), and IRF9 (**C**). Each promoter region spans 1,000 bp upstream and 500 bp downstream of the transcription start site. Predicted IRF1 binding affinity is displayed in purple using inverse log_2-transformed z-scores (2^z-score), and ChIP-seq coverage is shown in blue. Gene annotations are highlighted in green. These transcription factors were selected based on their altered expression in *IRF1* KO cells, as revealed by RNA-seq analysis.

A

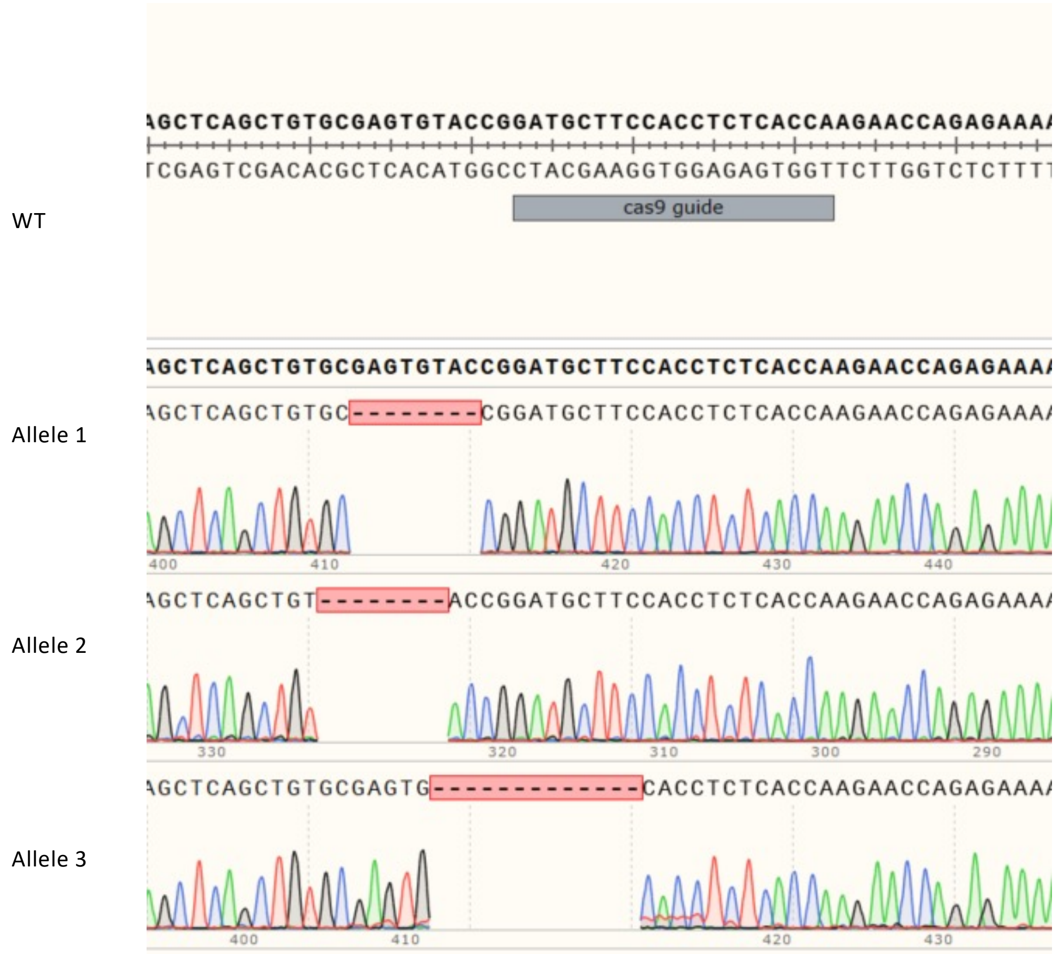

Figure S14. CRISPR/Cas9-mediated disruption of IRF1 in HeLa cells. (A) Genomic DNA sequencing of the CRISPR/Cas9 target region in the IRF1 locus in parental HeLa cells (WT) and the IRF1 knockout clone. The wild-type (WT) sequence and the three IRF1 alleles present in the HeLa clone (Allele 1–3) are shown aligned. Hyphens indicate deleted nucleotides relative to the WT sequence. All three alleles harbor indels at the CRISPR target site, consistent with disruption of the IRF1 coding sequence.
